# Whole genome sequence analysis of apparent treatment resistant hypertension status in participants from the Trans-Omics for Precision Medicine program

**DOI:** 10.3389/fgene.2023.1278215

**Published:** 2023-12-13

**Authors:** Nicole D. Armstrong, Vinodh Srinivasasainagendra, Farah Ammous, Themistocles L. Assimes, Amber L. Beitelshees, Jennifer Brody, Brian E. Cade, Yii-Der Ida Chen, Han Chen, Paul S. de Vries, James S. Floyd, Nora Franceschini, Xiuqing Guo, Jacklyn N. Hellwege, John S. House, Chii-Min Hwu, Sharon L. R. Kardia, Ethan M. Lange, Leslie A. Lange, Caitrin W. McDonough, May E. Montasser, Jeffrey R. O’Connell, Megan M. Shuey, Xiao Sun, Rikki M. Tanner, Zhe Wang, Wei Zhao, April P. Carson, Todd L. Edwards, Tanika N. Kelly, Eimear E. Kenny, Charles Kooperberg, Ruth J. F. Loos, Alanna C. Morrison, Alison Motsinger-Reif, Bruce M. Psaty, Dabeeru C. Rao, Susan Redline, Stephen S. Rich, Jerome I. Rotter, Jennifer A. Smith, Albert V. Smith, Marguerite R. Irvin, Donna K. Arnett

**Affiliations:** ^1^ Department of Epidemiology, University of Alabama at Birmingham, Birmingham, AL, United States; ^2^ Department of Biostatistics, University of Alabama at Birmingham, Birmingham, AL, United States; ^3^ Department of Epidemiology, School of Public Health, University of Michigan, Ann Arbor, MI, United States; ^4^ Survey Research Center, Institute for Social Research, Ann Arbor, MI, United States; ^5^ Division of Cardiovascular Medicine, Department of Medicine, Stanford University School of Medicine, Palo Alto, CA, United States; ^6^ Division of Endocrinology, Diabetes, and Nutrition, Program for Personalized and Genomic Medicine, Department of Medicine, University of Maryland School of Medicine, Baltimore, MD, United States; ^7^ Cardiovascular Health Research Unit, Department of Medicine, University of Washington, Seattle, WA, United States; ^8^ Division of Sleep and Circadian Disorders, Department of Medicine, Brigham and Women’s Hospital, Boston, MA, United States; ^9^ The Institute for Translational Genomics and Population Sciences, Department of Pediatrics, The Lundquist Institute for Biomedical Innovation at Harbor-UCLA Medical Center, Torrance, CA, United States; ^10^ Human Genetics Center, Department of Epidemiology, Human Genetics, and Environmental Sciences, School of Public Health, The University of Texas Health Science Center at Houston, Houston, TX, United States; ^11^ Center for Precision Health, School of Biomedical Informatics, The University of Texas Health Science Center at Houston, Houston, TX, United States; ^12^ Department of Medicine, University of Washington, Seattle, WA, United States; ^13^ Department of Epidemiology, University of Washington, Seattle, WA, United States; ^14^ Department of Epidemiology, Gillings School of Global Public Health, University of North Carolina, Chapel Hill, NC, United States; ^15^ Division of Genetic Medicine, Department of Medicine, Vanderbilt University Medical Center, Nashville, TN, United States; ^16^ Vanderbilt Genetics Institute, Vanderbilt University Medical Center, Nashville, TN, United States; ^17^ Biostatistics and Computational Biology Branch, National Institute of Environmental Health Sciences, Durham, NC, United States; ^18^ Section of Endocrinology and Metabolism, Department of Medicine, Taipei Veterans General Hospital, Taipei, Taiwan; ^19^ Division of Biomedical Informatics and Personalized Medicine, Department of Medicine, University of Colorado Anschutz Medical Campus, Aurora, CO, United States; ^20^ Department of Pharmacotherapy and Translational Research, College of Pharmacy, University of Florida, Gainesville, FL, United States; ^21^ Department of Medicine, University of Maryland, Baltimore, MD, United States; ^22^ Department of Epidemiology, Tulane University School of Public Health and Tropical Medicine, New Orleans, LA, United States; ^23^ The Charles Bronfman Institute for Personalized Medicine, Icahn School of Medicine at Mount Sinai, New York, NY, United States; ^24^ Department of Medicine, University of Mississippi Medical Center, Jackson, MS, United States; ^25^ Division of Epidemiology, Department of Medicine, Vanderbilt University Medical Center, Nashville, TN, United States; ^26^ Division of Nephrology, Department of Medicine, College of Medicine, University of Illinois Chicago, Chicago, IL, United States; ^27^ Institute for Genomic Health, Icahn School of Medicine at Mount Sinai, New York, NY, United States; ^28^ Division of Public Health Sciences, Fred Hutchinson Cancer Research Center, Seattle, WA, United States; ^29^ Division of Biostatistics, School of Medicine, Washington University in St. Louis, St. Louis, MO, United States; ^30^ Department of Public Health Sciences, Center for Public Health Genomics, University of Virginia, Charlottesville, VA, United States; ^31^ Center for Statistical Genetics, Department of Biostatistics, University of Michigan School of Public Health, Ann Arbor, MI, United States; ^32^ Office of the Provost, University of South Carolina, Columbia, SC, United States

**Keywords:** blood pressure, antihypertensive response, whole genome sequencing, TOPMed, treatment resistant hypertension

## Abstract

**Introduction:** Apparent treatment-resistant hypertension (aTRH) is characterized by the use of four or more antihypertensive (AHT) classes to achieve blood pressure (BP) control. In the current study, we conducted single-variant and gene-based analyses of aTRH among individuals from 12 Trans-Omics for Precision Medicine cohorts with whole-genome sequencing data.

**Methods:** Cases were defined as individuals treated for hypertension (HTN) taking three different AHT classes, with average systolic BP ≥ 140 or diastolic BP ≥ 90 mmHg, or four or more medications regardless of BP (*n* = 1,705). A normotensive control group was defined as individuals with BP < 140/90 mmHg (*n* = 22,079), not on AHT medication. A second control group comprised individuals who were treatment responsive on one AHT medication with BP < 140/ 90 mmHg (*n* = 5,424). Logistic regression with kinship adjustment using the Scalable and Accurate Implementation of Generalized mixed models (SAIGE) was performed, adjusting for age, sex, and genetic ancestry. We assessed variants using SKAT-O in rare-variant analyses. Single-variant and gene-based tests were conducted in a pooled multi-ethnicity stratum, as well as self-reported ethnic/racial strata (European and African American).

**Results:** One variant in the known HTN locus, *KCNK3*, was a top finding in the multi-ethnic analysis (*p* = 8.23E-07) for the normotensive control group [rs12476527, odds ratio (95% confidence interval) = 0.80 (0.74–0.88)]. This variant was replicated in the Vanderbilt University Medical Center’s DNA repository data. Aggregate gene-based signals included the genes *AGTPBP, MYL4, PDCD4, BBS9, ERG,* and *IER3*.

**Discussion:** Additional work validating these loci in larger, more diverse populations, is warranted to determine whether these regions influence the pathobiology of aTRH.

## 1 Introduction

Hypertension (HTN) is a leading risk factor for cardiovascular and renal disease (Global Burden of Metabolic Risk Factors for Chronic Diseases Collaboration, 2014). While awareness and treatment of HTN have improved, many patients experience suboptimal blood pressure (BP) control despite treatment (NCD Risk Factor Collaboration, 2019; Wijkman et al., 2021). Resistant HTN is defined as BP above target while using ≥3 antihypertensive (AHT) classes or the need to use ≥4 drug classes to achieve the BP goal (Carey et al., 2018; Akinyelure et al., 2020). Apparent treatment-resistant hypertension (aTRH) is used commonly in observational studies, where pseudo-resistant HTN cannot be excluded (e.g., caused by sub-optimal medication adherence, improper BP measurement, or white coat effect) (Judd and Calhoun, 2014; Akinyelure et al., 2020).

While the exact prevalence of aTRH is unknown, the estimated prevalence in population-based studies is between 12% and 15% among adults with HTN (Carey et al., 2018; Irvin et al., 2019) and even higher in clinical studies, which suggests aTRH affects about 15%–30% of patients with HTN (Hyman and Pavlik, 2001; Carey et al., 2018; Takahashi et al., 2021). Individuals with aTRH are at a higher risk of cardiovascular morbidity and mortality compared to individuals with controlled hypertension. Globally, an estimated 7.1 million deaths per year, as well as 62% of cerebrovascular disease and 49% of ischemic heart disease, are attributable to suboptimal BP control (systolic BP (SBP) > 115 mmHg) (Sarafidis and Bakris, 2008). The increased cardiovascular risk among patients with aTRH is likely due to the increased prevalence of concomitant co-morbidities such as diabetes, renal diseases, and obesity among persons with aTRH (Sarafidis and Bakris, 2008; Gupta et al., 2011) and is not exclusively due to high BP.

The etiology of aTRH is unknown, but is likely multifactorial (Vongpatanasin, 2014), with contributions of both behavioral/lifestyle and genetic factors suspected. While several large studies have identified genetic variants associated with HTN (Levy et al., 2009; Newton-Cheh et al., 2009; Franceschini et al., 2011; Evangelou et al., 2018; Giri et al., 2019), there are far fewer genetic studies of aTRH with limited findings (Lynch et al., 2013; Fontana et al., 2014; Dumitrescu et al., 2017; El Rouby et al., 2019; Irvin et al., 2019; Takahashi et al., 2021). We hypothesize that identifying the genetic contributors to aTRH may provide further insight into the etiology of the disease. The present study comprises 12 studies from the NHLBI Trans-Omics for Precision Medicine (TOPMed) program for a case-control single-variant and rare-variant, gene-based analyses of aTRH that utilizes TOPMed’s whole genome sequencing (WGS) data on over 29,000 participants (1,705 aTRH cases, 22,079 normotensive controls, and 5,424 controls who were treatment responsive). We attempted to validate our top variants in >10,000 participants from the International Consortium for Antihypertensive Pharmacogenomics Studies (ICAPS) and in >35,000 participants from Vanderbilt University Medical Center’s DNA repository, BioVU.

## 2 Methods

### 2.1 Discovery study population

For the current study, we conducted genomic analyses of aTRH among 29,208 participants from 12 TOPMed WGS studies (Supplementary Tables S1–S3). WGS and phenotype data were pooled across the studies for analysis. Additional information about the design for each study and the sampling of participants for WGS is available in the Supplementary Material. Informed consent was obtained from all participants, and the protocols for each study were approved by the institutional review board of the participating institutions.

### 2.2 Phenotype definition

AHT treatment data for each study was extracted by medication inventory or self-report, and medication classes that counted toward the total count in the present study are listed in Supplementary Table S4. Participants with conditions that may lead to secondary forms of HTN were excluded from analysis, including those with a body mass index (BMI) > 40 kg/m^2^ and/or an estimated glomerular filtration rate (eGFR) < 30 mL/min/1.73 m^2^, which defines advanced chronic kidney disease (Stages G4 and G5). Cases were defined as individuals taking three different AHT classes for HTN treatment who had an average systolic BP ≥ 140 mmHg or diastolic BP ≥ 90 mmHg or taking four or more medication classes regardless of BP level (total n = 1,705). A normotensive control group (n = 22,079) included individuals with systolic BP < 140 mmHg and diastolic BP < 90 mmHg and not reporting use of AHT medication. A second control group comprised of individuals who had BP controlled (BP < 140 mmHg systolic and <90 mmHg diastolic) on one AHT medication class (n = 5,424). The Seventh Report of the Joint National Committee on Prevention, Detection, Evaluation, and Treatment of High Blood Pressure (JNC 7) blood pressure guideline definitions were used in the current study given these guidelines were used for treatment thresholds during the time the data was collected for the majority of studies included in the analysis (Chobanian et al., 2003). Phenotypes were harmonized across studies using a protocol developed by the TOPMed Blood Pressure Working Group and have been previously described (Kelly et al., 2022).

### 2.3 Whole genome sequencing (WGS) and quality control (QC)

A detailed description of the TOPMed WGS methods has been reported previously (Taliun et al., 2021). Briefly, WGS was performed at an average depth of >30x by six sequencing centers (Broad Genomics, Northwest Genome Center, Illumina, New York Genome Center, Baylor, and McDonnell Genome Institute) with Illumina HiSeq X10 technology (Mikhaylova et al., 2021). Genotype calling was conducted jointly across the TOPMed studies using the GotCloud pipeline (Jun et al., 2015). Variants were removed from analysis with excess heterozygosity or Mendelian inconsistencies, with overall missingness greater than 5%, or with overlapping centromeric regions. Genotypes with average read depth less than 10x were set to missing. Samples that were duplicated, had sex discrepancies, were misidentified or had consent issues, or had poor concordance across WGS and array data were excluded, as previously described (Kelly et al., 2022).

### 2.4 Statistical analysis

Multi-ethnic and race/ethnicity-specific (self-reported African American [AA] and self-reported European American [EA]) analyses were conducted and baseline demographic information is presented in Table 1. In single variant analyses, single-nucleotide variants (SNVs) and insertion-deletion (indel) variants with a minor allele frequency (MAF) ≥0.5% were retained. This threshold was chosen to leverage rare variants observed in WGS (a lower filter than generally considered by GWAS) but we did not include rarer variants due to limited case counts, especially in the self-reported race strata. For the SNV association analysis, we used a logistic mixed model in Scalable and Accurate Implementation of Generalized mixed models (SAIGE) software (Zhou et al., 2018). We adjusted for familial relationships by constructing a genetic relationship matrix as well as age, sex, and ancestry principal components. Ancestry principal components were generated by the TOPMed Data Coordinating Center in PC-AiR (Conomos et al., 2015), which is available in the GENESIS R package A saddle-point approximation was conducted to account for case-control imbalance in the analyses. A Bonferroni correction was applied to correct for multiple testing. The statistical significance threshold was set at *p* < 5.00E-08. As a secondary analysis, we implemented inverse variance-weighted, fixed effects meta-analysis on the summary statistics from the self-reported AA and EA strata for (Global Burden of Metabolic Risk Factors for Chronic Diseases Collaboration, 2014) aTRH versus normotensive controls and (NCD Risk Factor Collaboration, 2019) aTRH versus treatment-responsive controls, using METAL software (Willer et al., 2010). Statistical heterogeneity was evaluated using Cochran’s chi-square test in METAL.

**TABLE 1:** Baseline characteristics of study participants.

	aTRH cases	Treatment-responsive controls	Normotensive controls
N	1,705	5,424	22,079
Age, years	64.67 ± 10.44	60.80 ± 10.75	54.79 ± 13.33
Self-reported race/ethnicity, n (%)			
African	768 (45.04%)	1,924 (35.47%)	5,032 (22.79%)
Asian	51 (2.99%)	236 (4.35%)	2,478 (11.22%)
European	395 (23.17%)	2,730 (50.33%)	12,483 (56.54%)
Hispanic	448 (26.28%)	477 (8.79%)	1,844 (8.35%)
Other/Unknown	43 (2.52%)	57 (1.05%)	242 (1.10%)
Female, n (%)	1,039 (60.94%)	3,855 (71.07%)	14,141 (64.05%)
BMI, kg/m^2^	29.99 ± 4.75	28.48 ± 4.77	26.55 ± 4.59
eGFR, mL/min/1.73m^2^	71.21 ± 21.74	86.81 ± 21.24	92.00 ± 20.21
SBP, mmHg	164.27 ± 23.29	135.80 ± 12.31	116.33 ± 12.50
DBP, mmHg	88.98 ± 13.01	81.32 ± 9.27	70.39 ± 8.88
AHT Medication Use, n (%)			
Angiotensin-converting enzyme inhibitor (ACE-I)	959 (56.25%)	877 (16.17%)	0 (0%)
Aldosterone antagonist (MRA)	255 (14.96%)	46 (0.85%)	0 (0%)
Alpha-blocker (AB)	487 (28.56%)	75 (1.38%)	0 (0%)
Angiotensin receptor blocker (ARB)	385 (22.58%)	202 (3.72%)	0 (0%)
Beta-blocker (BB)	1,153 (67.62%)	1,097 (20.22%)	0 (0%)
Calcium channel blocker (CCB)	1,024 (60.06%)	1,052 (19.40%)	0 (0%)
Central acting agent (CAA)	225 (13.20%)	92 (1.70%)	0 (0%)
Diuretic	1,389 (81.47%)	1,801 (33.20%)	0 (0%)
Direct vasodilator (DV)	205 (12.02%)	319 (5.88%)	0 (0%)

Abbreviations: aTRH- apparent treatment-resistant hypertension; BMI- body mass index; eGFR-estimated glomerular filtration rate; SBP- systolic blood pressure; DBP- diastolic blood pressure; AHT-antihypertensive.

Combined burden and variance-component tests (i.e., omnibus sequence kernel association tests, SKAT-O) were applied to examine the association between aTRH status and aggregate rare variants in Efficient and Parallelizable Association Container Toolbox (EPACTS) software (EPACTS, 2019). Variants were functionally annotated using the Whole Genome Sequence Annotator (WGSA) (Liu et al., 2016) and classified as high or moderate impact (Supplementary Table S5). Generalized linear mixed models parallel to the single variant association tests were conducted, adjusting for age, sex, and ancestry principal components. Aggregate results were retained if there were i) 2-4 SNVs, none of which were singletons, or ii) ≥5 SNVs comprising the gene region. The gene-based signals with *p* < 2.50E-06 (=0.05/20,000 gene regions) and *p* < 1.00E-04 achieved statistical or suggestive significance, respectively.

### 2.5 Look-up validation in BioVU and ICAPS

We sought replication using existing data in 5,729 AA and 31,805 EA participants from Vanderbilt University Medical Center’s DNA Repository (BioVU), as well as 10,801 participants comprising the International Consortium of Antihypertensive Pharmacogenomic Studies (ICAPS) aTRH-specific study (2,190 AA, 8,074 EA, and 537 Hispanic). Briefly in BioVU, aTRH status was defined through the presence of an HTN ICD-9 or ICD-10 code, treatment with an AHT, or having two outpatient, non-emergency department SBP >140 mmHg and/or two diastolic BP (DBP) measures >90 mmHg. Patients with aTRH were identified based on failure to achieve controlled BP on three AHTs, including a thiazide diuretic, or prescribed four or more medications regardless of achieving control. The comparison group of hypertensive patients who achieved BP control on one or two medications, excluding participants with chronic kidney disease (stage 4 and 5), or those with secondary causes of HTN. A second control group consisted of individuals without HTN as defined above (Shuey et al., 2018). In the ICAPS aTRH analysis, aTRH was defined as uncontrolled HTN using three or more AHTs from different classes (BP ≥ 140/90 mmHg) or controlled HTN on four or more AHT medications. Treatment-responsive controls were individuals with controlled BP (<140/90) with the use of three or fewer AHTs. Quality control and analyses were performed at the cohort level for the five randomized controlled clinical trials, and then meta-analyzed (combined and race-stratified). Variant lookups were performed for both replication cohorts based on the top findings presented in Table 2. Replication significance was determined as *p* < 1.39E-03 (=0.05/36 unique genetic loci).

**TABLE 2:** Top ten variants observed for association analysis of aTRH in each self-reported race/ethnicity-control strata.

rsID	CHR:BP	REF/EA	EAF	Or (95% CI)	*p*-value^a^	Consequence	Gene(s)
Multi-ethnicity: aTRH (n = 1,705) versus treated controls (n = 5,424)							
rs1175395756	chr6:124515703	TTC​GCT​TTC​TCT​CCG​TC/T	0.2586	0.35 (0.25, 0.51)	1.38E-08	intronic	*NKAIN2*
rs6086545	chr20:935220	C/A	0.1712	1.35 (1.20, 1.52)	4.86E-07	intergenic	*ANGPT4;RSPO4*
rs189036218	chr6:8077088	C/T	0.0054	3.73 (2.23, 6.25)	5.33E-07	ncRNA_intronic	*EEF1E1-BLOC1S5*
rs538121738	chr3:84401497	G/A	0.0104	2.51 (1.73, 3.65)	1.25E-06	intergenic	*LINC02008;LINC00971*
rs116250062	chr5:108665213	T/A	0.0065	4.14 (2.33, 7.35)	1.29E-06	intergenic	*FBXL17;LINC01023*
rs138450763	chr6:8129403	G/A	0.0052	3.64 (2.16, 6.14)	1.34E-06	intergenic	*EEF1E1-BLOC1S5;SLC35B3*
rs12335619	chr9:3669438	G/C	0.0497	0.64 (0.54, 0.77)	1.48E-06	ncRNA_intronic	*RFX3-AS1*
rs140823205	chr12:103789678	T/TA	0.0270	0.57 (0.45, 0.72)	1.59E-06	intronic	*NT5DC3*
rs1546149	chr8:15475599	G/A	0.2864	1.26 (1.14, 1.38)	1.64E-06	intergenic	*SGCZ;TUSC3*
rs761571	chr20:932022	T/C	0.1880	1.31 (1.17, 1.47)	1.89E-06	intergenic	*ANGPT4;RSPO4*
Multi-ethnicity: aTRH (n = 1,705) versus normotensive controls (n = 22,079)							
rs1175395756	chr6:124515703	TTC​GCT​TTC​TCT​CCG​TC/T	0.3455	0.32 (0.23, 0.43)	3.83E-13	intronic	*NKAIN2*
rs142718434	chr18:69043215	C/T	0.0065	2.56 (1.78, 3.68)	3.98E-07	intronic	*CCDC102B*
rs74574593	chr21:36426260	G/A	0.0183	1.74 (1.40, 2.17)	6.77E-07	intergenic	*CHAF1B;CLDN14*
rs61312821	chr5:160927044	G/A	0.1129	1.42 (1.23, 1.63)	7.51E-07	intergenic	*ATP10B;LINC02159*
rs12476527	chr2:26692756	G/T	0.4896	0.80 (0.74, 0.88)	8.23E-07	UTR5	*KCNK3*
rs79890489	chr3:27831750	C/A	0.0059	2.47 (1.72, 3.55)	1.05E-06	ncRNA_exonic	*LINC01981*
rs115299786	chr3:27830202	T/G	0.0062	2.43 (1.70, 3.48)	1.18E-06	ncRNA_intronic	*LINC01980*
rs191754301	chr5:26402669	G/A	0.0065	2.40 (1.68, 3.41)	1.20E-06	intergenic	*LINC02211;CDH9*
rs899934113	chr3:27845147	TA/T	0.0062	2.42 (1.69, 3.47)	1.26E-06	intronic	*LINC01980*
rs76818748	chr15:54329458	T/C	0.0586	0.63 (0.52, 0.76)	1.46E-06	intronic	*UNC13C*
AFR: aTRH (n = 768) versus treated controls (n = 1,924)							
rs1546149	chr8:15475599	G/A	0.3398	1.45 (1.27, 1.65)	2.73E-08	intergenic	*SGCZ;TUSC3*
rs183165107	chr5:38365438	G/A	0.0100	5.48 (2.90, 10.36)	1.59E-07	intronic	*EGFLAM*
rs80116392	chr11:133314740	G/C	0.0721	1.87 (1.48, 2.37)	2.06E-07	intronic	*OPCML*
rs566745771	chr12:132295343	C/T	0.0050	10.27 (4.20, 25.12)	3.28E-07	intronic	*GALNT9*
rs74179759	chr20:15137285	G/A	0.0063	7.83 (3.51, 17.45)	4.78E-07	intronic	*MACROD2*
rs6595479	chr5:123821788	G/A	0.0520	2.02 (1.53, 2.66)	7.60E-07	intergenic	*CSNK1G3;LINC01170*
rs76368370	chr18:71114951	C/G	0.0396	2.21 (1.62, 3.03)	7.68E-07	intergenic	*GTSCR1;LINC01541*
rs116645995	chr1:239428421	C/T	0.0137	3.78 (2.23, 6.40)	7.87E-07	intronic	*CHRM3*
rs17126926	chr10:109992693	T/C	0.0555	1.98 (1.51, 2.60)	9.83E-07	ncRNA_intronic	*ADD3-AS1*
rs115632010	chr4:93349186	A/T	0.0165	3.48 (2.11, 5.74)	9.99E-07	intronic	*GRID2*
AFR: aTRH (n = 768) versus normotensive controls (n = 5,032)							
rs62434735	chr6:147600061	G/A	0.0569	2.06 (1.58, 2.68)	7.45E-08	intergenic	*SAMD5;SASH1*
rs183634807	chr2:32050050	G/A	0.0138	3.88 (2.33, 6.47)	1.92E-07	intergenic	*DPY30;SPAST*
rs11963682	chr6:147594975	C/T	0.0555	2.00 (1.54, 2.61)	2.85E-07	intergenic	*SAMD5;SASH1*
rs117043225	chr8:56731207	C/T	0.0054	8.78 (3.75, 20.57)	5.71E-07	intergenic	*LINC00968;IMPAD1*
rs73987603	chr2:212980098	G/A	0.1961	0.68 (0.59, 0.79)	6.68E-07	intergenic	*MIR4776-2;IKZF2*
rs113287654	chr12:127301710	G/C	0.0761	1.75 (1.40, 2.20)	9.14E-07	intergenic	*LINC02376;LINC02375*
rs11017247	chr10:130427166	A/T	0.3075	0.74 (0.65, 0.83)	1.09E-06	intergenic	*GLRX3;MIR378C*
rs190813229	chr2:32006833	C/T	0.0115	3.97 (2.28, 6.91)	1.10E-06	intronic	*MEMO1*
rs115965650	chr2:226205376	C/T	0.0070	7.53 (3.34, 16.97)	1.12E-06	intergenic	*LOC646736;MIR5702*
rs74552450	chr2:226251404	T/A	0.0070	6.93 (3.17, 15.16)	1.23E-06	intergenic	*LOC646736;MIR5702*
EUR: aTRH (n = 395) versus treated controls (n = 2,730)							
rs11711860	chr3:38776013	C/A	0.2566	1.64 (1.37, 1.97)	6.16E-08	intronic	*SCN10A*
rs73064548	chr3:38782346	C/G	0.2567	1.64 (1.37, 1.97)	6.18E-08	intronic	*SCN10A*
rs62244107	chr3:38778263	C/T	0.2566	1.64 (1.37, 1.96)	6.30E-08	intronic	*SCN10A*
rs62244108	chr3:38781988	G/A	0.2567	1.64 (1.37, 1.96)	6.34E-08	intronic	*SCN10A*
rs62244106	chr3:38777782	T/C	0.2570	1.64 (1.37, 1.96)	6.54E-08	intronic	*SCN10A*
rs7430726	chr3:38776830	T/C	0.2570	1.64 (1.37, 1.96)	6.54E-08	intronic	*SCN10A*
rs11129808	chr3:38785608	T/C	0.2571	1.64 (1.37, 1.96)	7.83E-08	intronic	*SCN10A*
rs12172966	chr3:38785717	T/G	0.2570	1.63 (1.36, 1.95)	1.08E-07	intronic	*SCN10A*
rs11927856	chr3:38779283	T/C	0.2586	1.62 (1.36, 1.94)	1.11E-07	intronic	*SCN10A*
rs4072090	chr3:38773939	G/T	0.2574	1.62 (1.35, 1.94)	1.35E-07	intronic	*SCN10A*
EUR: aTRH (n = 395) versus normotensive controls (n = 12,483)							
rs62244107	chr3:38778263	C/T	0.2532	1.54 (1.30, 1.82)	5.64E-07	intronic	*SCN10A*
rs11711860	chr3:38776013	C/A	0.2532	1.54 (1.30, 1.82)	5.73E-07	intronic	*SCN10A*
rs62244108	chr3:38781988	G/A	0.2533	1.54 (1.30, 1.82)	6.02E-07	intronic	*SCN10A*
rs7430726	chr3:38776830	T/C	0.2534	1.54 (1.30, 1.82)	6.24E-07	intronic	*SCN10A*
rs73064548	chr3:38782346	C/G	0.2534	1.54 (1.30, 1.82)	6.31E-07	intronic	*SCN10A*
rs62244106	chr3:38777782	T/C	0.2534	1.54 (1.30, 1.82)	6.35E-07	intronic	*SCN10A*
rs11129808	chr3:38785608	T/C	0.2536	1.53 (1.30, 1.81)	6.76E-07	intronic	*SCN10A*
rs34623024	chr10:9717155	T/G	0.2002	1.58 (1.32, 1.89)	8.42E-07	intergenic	*LOC101928272;LOC101928298*
rs12779933	chr10:9715550	C/T	0.1994	1.58 (1.32, 1.89)	8.60E-07	intergenic	*LOC101928272;LOC101928298*
rs12172966	chr3:38785717	T/G	0.2534	1.53 (1.29, 1.81)	8.67E-07	intronic	*SCN10A*

^a^ *p*-value using saddle point approximation. Statistical significance *p* < 5.00E-08 (*p*-value bolded).

Abbreviations: rsID-reference SNP, cluster ID; CHR-chromosome; BP- base position hg38 build; REF- reference allele; EA-effect allele; EAF- effect allele frequency; OR-odds, ratio; CI- confidence interval; aTRH- apparent treated-resistant hypertension; UTR5- 5′ untranslated region; ncRNA-non-coding RNA; AFR-self-reported African American race/ethnicity; EUR-self-reported European American race/ethnicity.

## 3 Results

### 3.1 Study characteristics

The baseline characteristics of the study participants are shown in Table 1. Across the 12 TOPMed cohorts, 1,705 aTRH cases were compared to i) 5,424 treatment-responsive controls and ii) 22,079 normotensive controls. On average, aTRH cases (64.67 years) were older than treatment-responsive (60.80 years) and normotensive (54.79 years) controls and were more likely to be AA (45.04% cases). Cases also presented, on average, with a higher BMI (29.99 kg/m^2^) compared with treatment-responsive (28.48 kg/m^2^) and normotensive (26.55 kg/m^2^) controls, as well as lower glomerular filtration rate compared to treatment-responsive and normotensive controls (71.21 mL/min/1.73 m^2^ versus 86.81 mL/min/1.73 m^2^ and 92.00 mL/min/1.73 m^2^, respectively), though on average still in the normal range (Table 1).

### 3.2 Single-variant analysis

The top 10 results for each case-control model are presented in Table 2. When comparing aTRH cases to normotensive controls, the top finding for the pooled, multi-race/ethnicity analysis was rs1175395756, an indel variant located in the sodium/potassium transporting ATPase interacting 2 (*NKAIN2*) gene. For rs1175395756, the odds of having aTRH were lower among those with the deletion (odds ratio [OR] = 0.32, 95% confidence interval [95% CI] = 0.23–0.43, *p* = 3.83E-13, effect allele frequency [EAF] = 0.27). Further, rs12476527, located in the 5’ untranslated region of the potassium two pore domain channel subfamily K member 3 gene (*KCNK3*), resulted in lower odds of aTRH (OR [95% CI] = 0.80 [0.74–0.88], *p* = 8.23E-07) for those with an effect allele T. When comparing the aTRH cases to 5,424 treatment-responsive controls, the effect of rs1175395756 was consistent (OR [95% CI] = 0.35 [0.25–0.51], *p* = 1.38E-08, EAF = 0.35) with the analysis of normotensive controls. Further, the effect alleles of 11 intergenic variants located on chromosome 20 between the angiopoietin-4 (*ANGPT4*) and R-spondin 4 (*RSPO4*) genes (EAF ranging from 0.15–0.18) had increased odds of aTRH (OR [95% CI] = 1.35 [1.20–1.52], *p* = 4.86E-07 for the lead SNV rs6086545).

Among the EAs, the top findings for 395 aTRH cases compared to both control groups were intronic to the sodium voltage-gated channel alpha subunit 10 (*SCN10A*) gene. All 13 of the suggestive variants observed for the normotensive control group comparison were also associated with aTRH for the treatment-responsive control group comparison (Table 2 and Supplementary Tables S6, S7) with *p* ≤ 2.36E-06. The majority of the suggestive *SCN10A* variants were in strong LD (*r* ^2^ > 0.8; Supplementary Table S8). The only statistically significant finding for the single variant analysis in the African-ethnicity stratum was an intergenic variant (rs1546149) between the zeta-sarcoglycan gene (*SGCZ*) and the tumor suppressor candidate 3 gene (*TUSC3*) on chromosome 8 (Table 2). All SNV results that met the suggestive statistical significance threshold *p* < 1.00E-05 in each ethnic strata and control group comparison can be found in Supplementary Tables S6, S7, S9–S12. While we observed some overlap by control group analysis in ethnic strata, there was limited overlap across race/ethnicity-specific analyses. Manhattan and QQ plots for each analysis are presented in Supplementary Figures S1–S6, and there was no evidence for deviation of *p*-values from their expected values (Supplementary Table S13). In a secondary meta-analysis of the AA and EA strata results, we did not observe any statistically significant findings. rs1546149 had nominal significance for the treatment-responsive control group (*p* = 7.31E-08) (Supplementary Tables S14, S15; Supplementary Figures S7, S8).

Upon variant look-up in BioVU and ICAPS, rs12476527 located in *KCNK3* was replicated in the BioVU EA aTRH versus normotensive control analysis with the same direction of effect (OR [95% CI] = 0.88 [0.82–0.95], *p* = 6.10E-04) (Supplementary Table S16). While not statistically significantly associated with aTRH in our other analyses, the variant had a consistent direction of effect in all our analyses, with marginally significant *p*-values for the multi-ethnic aTRH versus treated controls (OR [95% CI] = 0.88 [0.80–0.96], *p* = 5.15E-03), AA aTRH versus normotensive controls (OR [95% CI] = 0.83 [0.72–0.95], *p* = 8.47E-03), and EA aTRH versus normotensive controls (OR [95% CI] = 0.81 [0.69–0.94], *p* = 5.81E-03). Likewise, while this variant was not significant in the BioVU AA lookup, a consistent direction of effect (OR [95% CI] = 0.82 [0.68–0.98], *p* = 3.18E-02) was observed. Since rs12476527 was originally discovered in our analysis of aTRH cases versus normotensive controls, we did not look this variant up in the ICAPS replication cohort, which included treatment-responsive controls only.

### 3.3 Gene-based, rare-variant analysis

Gene-based results are presented in Table 3 (*p* < 2.5E-06). Expanded results for genes with suggestive significance are presented in Supplementary Tables S17–S19. Biologically plausible signals included the genes ATP/GTP binding carboxypeptidase 1 (*AGTPBP1*), myosin light chain 4 (*MYL4*), programmed cell death 4 (*PDCD4*), Bardet-Biedel Syndrome 9 (*BBS9*), erythroblast transformation-specific transcription factor ERG (*ERG*), and immediate early response 3 (*IER3*). In the multi-ethnic aggregate analysis of high-impact variants, *AGTPBP1* was associated with aTRH in comparison to normotensive controls (5 variants, 4 singletons *p* = 3.04E-07). Among AA participants, the aggregate of 20 total variants (including 10 singletons) of high-moderate impact in the *MYL4* gene region, was associated with aTRH in comparison to the normotensive control group (*p* = 8.83E-07). Also, in this stratum, the aggregate of five total high-impact variants (three singletons) in the *PDCD4* gene region was associated (*p* = 1.59E-06) with case status when compared to the normotensive control group. Three gene regions of interest were observed in the EA analysis including *BBS9* (high impact; normotensive controls; *p* = 1.29E-14), *ERG* (high-moderate impact; normotensive controls; *p* = 1.03E-10), and *IER3* (high-moderate impact; *p* = 2.48E-07 and *p* = 2.84E-07 for normotensive and treatment responsive controls, respectively).

**TABLE 3:** Statistically significant (*p* < 2.50E-06) gene-based results.

CHR:BP (hg38)	Gene	Total variants	Singletons	*p*-value	Control group	Functional impact^a^
Multi-ethnicity						
chr7:102573812-102616680	*RASA4*	5	4	2.44E-11	Normotensive	High-Moderate
chr11:617593-624900	*CDHR5*	6	4	2.93E-07	Normotensive	High
chr9:85547252-85741961	*AGTPBP1*	5	4	3.04E-07	Normotensive	High
chr20:44910144-44958409	*PABPC1L*	5	2	5.66E-07	Normotensive	High
chr3:157146572-157160085	*CCNL1*	51	34	7.75E-07	Normotensive	High-Moderate
chr20:35616035-35620978	*SPAG4*	6	2	1.23E-06	Normotensive	High
chr5:151016922-151017411	*AC008641.1*	2	0	2.16E-06	Normotensive	High-Moderate
AFR						
chr17:47209444-47223029	*MYL4*	20	10	8.83E-07	Normotensive	High-Moderate
chr10:110876746-110896067	*PDCD4*	5	3	1.59E-06	Normotensive	High
EUR						
chr7:33152724-33605225	*BBS9*	5	5	1.29E-14	Normotensive	High
chr19:2515043-2520681	*GNG7*	6	6	4.55E-13	Normotensive	High-Moderate
chr10:122454730-122456931	*ARMS2*	7	7	5.33E-12	Normotensive	High-Moderate
chr22:37971252-37983774	*SOX10*	11	9	3.60E-11	Treated	High-Moderate
chr21:38383429-38575700	*ERG*	28	23	1.03E-10	Normotensive	High-Moderate
chr6:31960118-31968877	*SKIV2L*	6	4	3.46E-10	Normotensive	High
chr3:150660005-150703898	*ERICH6*	5	5	5.27E-10	Normotensive	High
chr1:153070631-153070827	*SPRR2B*	5	2	1.32E-09	Normotensive	High-Moderate
chr16:87398822-87403097	*MAP1LC3B*	5	5	9.13E-09	Normotensive	High
chr22:37971252-37983774	*SOX10*	29	18	9.81E-09	Normotensive	High-Moderate
chr11:72216900-72221653	*FOLR2*	8	4	4.65E-08	Normotensive	High
chr19:42753372-42855618	*PSG8*	8	6	7.53E-08	Normotensive	High
chr12:48830188-48844031	*DDX23*	8	7	1.16E-07	Normotensive	High
chr9:128371381-128389729	*URM1*	23	18	1.28E-07	Normotensive	High-Moderate
chr3:50347615-50350643	*NPRL2*	27	17	2.09E-07	Normotensive	High-Moderate
chr6:159906956-159907931	*MAS1*	2	0	2.47E-07	Normotensive	High
chr6:30743938-30744514	*IER3*	14	10	2.48E-07	Normotensive	High-Moderate
chr6:30743938-30744514	*IER3*	7	5	2.84E-07	Treated	High-Moderate
chr16:56638740-56639943	*MT1A*	7	7	3.14E-07	Normotensive	High-Moderate
chr1:225887081-225888019	*RP4-559A3.7*	6	5	4.05E-07	Normotensive	High-Moderate
chr1:43307827-43321659	*TIE1*	5	4	4.74E-07	Normotensive	High
chr5:155013860-155017553	*KIF4B*	9	8	5.63E-07	Normotensive	High
chr3:10034704-10101203	*FANCD2*	5	3	7.28E-07	Normotensive	High
chr1:54609269-54619933	*FAM151A*	5	2	7.35E-07	Normotensive	High
chr19:2785665-2813226	*THOP1*	30	19	7.58E-07	Normotensive	High
chr5:151016922-151017411	*AC008641.1*	2	0	1.17E-06	Normotensive	High-Moderate
chr13:48342667-48476805	*RB1*	5	4	1.19E-06	Treated	High
chr22:21383992-21388771	*RIMBP3B*	5	5	1.35E-06	Normotensive	High-Moderate
chr11:94429946-94492670	*MRE11A*	27	20	1.40E-06	Normotensive	High
chr12:48342998-48350059	*ZNF641*	5	5	1.57E-06	Normotensive	High
chr20:5127096-5190229	*CDS2*	23	15	2.04E-06	Normotensive	High-Moderate
chr1:209617505-209650942	*LAMB3*	8	6	2.08E-06	Normotensive	High
chr2:74530009-74532941	*HTRA2*	6	4	2.33E-06	Normotensive	High
chr17:40074515-40093367	*THRA*	27	20	2.36E-06	Normotensive	High-Moderate
chr3:111999445-112013701	*TAGLN3*	17	12	2.37E-06	Normotensive	High-Moderate

^a^ Definition of high versus high-moderate functional impact can be found in Supplementary Table S5.

Abbreviations: AFR-self-reported African American race/ethnicity; EUR-self-reported European American race/ethnicity.

## 4 Discussion

In the current study utilizing sequencing data from over 29,000 participants from TOPMed (26.44% AAs, 9.47% Asian, 53.44% EA, 9.48% Hispanic ethnicity, 1.17% other or unknown race/ethnicity), we investigated the genetic contributors to aTRH. Analyses were performed in a pooled, multi-ethnic analysis, as well as in self-reported AA and EA racial/ethnic strata. We identified three variants at the single-variant level and 44 gene regions that exceeded statistical significance for association with aTRH.

One of our top findings was located in the *KCNK3* gene, which encodes a member of the superfamily of potassium channel proteins and was replicated in the BioVU data (Gene, 2004). *KCNK3* has been previously linked to HTN in genetic studies. In a multi-racial/ethnic analysis of >7,800 individuals of European, African, Hispanic, and Chinese ethnicity within the Multi-Ethnic Study of Atherosclerosis (MESA) study, several *KCNK3* variants were associated with measures of BP (including SBP, DBP, mean arterial pressure, and pulse pressure) across race/ethnic groups. Additionally, this study showed in a rodent model, that genetic disruption of *KCNK3* produces hyperaldosteronism (Manichaikul et al., 2016). More recently, loss-of-function *KCNK3* mutations have been identified as likely causative in heritable pulmonary arterial HTN (Ma et al., 2013; West et al., 2021). Specifically, rs12476527 has been associated with both pulse pressure (*p* = 2.55E-21) and SBP (*p* = 5.89E-50) across multi-ethnic meta-analyses (Giri et al., 2019).

A single-variant intronic to *NKAIN2* was associated with aTRH in both control strata in the multi-ethnic analysis. *NKAIN2* is a transmembrane protein that interacts with the beta subunit of a sodium/potassium transporting ATPase (Gene, 2004). Previous studies have shown that *NKAIN2* may be related to neurological phenotypes, such as schizophrenia and bipolar disease (Blokland et al., 2022), as well as being a susceptibility locus for BMI (Yasukochi et al., 2018). This variant has not been directly associated with HTN to our knowledge, though a 2016 study reported a different SNV (not in LD with rs1175395756) in *NKAIN2* (rs332607) was associated with pubertal SBP and adult SBP in the International Consortium of Blood Pressure (Parmar et al., 2016). Of note, the sodium/potassium transporting ATPase-s are ubiquitously expressed. In the heart they influence cell calcium and contractility by generating the Na + gradient driving Ca++ out of the cell via the Na+/Ca++ exchanger. They are also receptors for cardiac glycosides such as digitalis (a heart failure and antiarrhythmic drug) which increases cardiac contractility (McDonough et al., 2002). Since most AHTs act on the kidneys, heart and vasculature it is difficult to directly link *NKAIN2* (primarily expressed in the brain) to AHT treatment response. However, some AHTs do influence the sympathetic nervous system (e.g., alpha blockers, centrally acting agents, and beta blockers) which could potentially link a mechanism to this gene, though that is beyond the scope of our study.

An additional intergenic region between *ANGPT4* and *RSPO4* was identified in our multi-ethnic analysis utilizing the treatment-responsive controls. *ANGPT4* encodes an angiopoietin shown to be involved in vascular development (Gene, 2004), angiogenesis, and vascular disease (Abu-Farha et al., 2018; Ali et al., 2022). *RSPO4* is a member of the R-spondin family of proteins that have emerged as important regulators of WNT signaling (Jin and Yoon, 2012). There is evidence for Wnt’s role in the regulation of BP and the pathogenesis of HTN (Abou Ziki and Mani, 2017). Lastly, and convincing in our European-specific analysis, 15 intronic variants of *SCN10A* met or exceeded our suggestive significance threshold (*p* < 1.00E-05). *SCN10A* encodes the voltage-gated sodium channel, Nav1.8, and is flanked by *SCN5A* and *SCN11A*, both of which encode for voltage-gated sodium channels, Nav1.5 and Nav1.9, respectively (Macri et al., 2018). *SCN10A* is expressed in the myocardium and preferentially in the specialized Purkinje fibers of the cardiac conduction system (Sotoodehnia et al., 2010). Several genome-wide association studies have reported that SCN10A is involved in heart rate and dysrhythmia (Chambers et al., 2010; Holm et al., 2010; Pfeufer et al., 2010; Sotoodehnia et al., 2010; Liu et al., 2020). While the association between SCN10A and cardiac conduction is established making this gene especially interesting in our study of aTRH, *SCN10A* and other voltage gated sodium channels are not AHT drug targets. However, voltage gated sodium channels are important drug targets for other disorders (e.g., seizures, cardiac arrhythmias) (Wisedchaisri and Gamal El-Din, 2022) and further investigation of how different AHTs may indirectly affect their action is warranted (Liu et al., 2020). Most of our novel findings were derived from our aggregate, rare-variant (gene-based) analysis, which was to be expected due to the high coverage of rare genetic variants through TOPMed sequencing. In the multi-ethnic analysis, the gene region on chromosome 9 spanning the *AGTPBP1* gene was of interest due to its previous association with cardiometabolic-related traits. *AGTPBP1 is a* metallocarboxypeptidase that mediates protein deglutamylation of tubulin and non-tubulin target proteins (Gene, 2004) and has been associated with BMI (Yasukochi et al., 2018), as well as increased blood pressure in spontaneously hypertensive rat lines (Bell et al., 2011).

An additional two gene regions were associated with aTRH in the AA stratum. *MYL4*, located on chromosome 17, and *PDCD4*, located on chromosome 10, both exceeded the significance threshold for the aTRH-normotensive case-control analysis. *MYL4* encodes a myosin alkali light chain that is found in embryonic muscle and adult atria (Gene, 2004). Rs117154502, an intronic variant of *MYL4,* has been associated with both pulse pressure and SBP among Europeans (Plotnikov et al., 2022). *PDCD4,* the other gene region that was identified in the AA stratum, is a tumor suppressor gene that encodes a protein that binds to eukaryotic translation initiation factor 4A1 (Gene, 2004). *PDCD4* is a direct target of miR-532-5p, a microRNA that has a potential function in cardiomyocyte response to hypoxia (Jin et al., 2018). *BBS9, ERG,* and *IER3* were all gene regions implicated with aTRH in the European stratum. *BBS9* is a gene associated with Bardet-Biedel Syndrome (BBS), an autosomal recessive condition with multiple clinical features including renal abnormalities, obesity, and HTN (Zhao and Rahmouni, 2022). *ERG* encodes a member of the erythroblast transformation-specific family of transcription factors (Gene, 2004). Recently, ERG has emerged as a major regulator of endothelial function (Shah et al., 2016; Kalna et al., 2019), and is important in the regulation of tissue-specific processes that include hematopoiesis, angiogenesis, and vascular inflammation (Seth and Watson, 2005; Shah et al., 2016). Of note, our variants comprising this gene region are not in LD with a previously reported *ERG* variant (rs117870289) associated with DBP (Giri et al., 2019). *IER3* may play a role in the ERK signaling pathway by inhibiting the dephosphorylation of ERK (Gene, 2004). Animal studies have shown results that point to a cardiovascular role of IER related to blood pressure (De Keulenaer et al., 2002; Arlt and Schafer, 2011).

As one of the largest WGS analyses on aTRH, this study has several strengths. First, by leveraging the TOPMed consortium data, we were able to interrogate both common and rare variants in a racially/ethnically diverse sample. This was facilitated by the TOPMed Blood Pressure Working Group, which harmonized phenotypes and related variables in a pooled dataset across observational cohorts to pair with the pooled genomic data. Our study is not without limitations. Although it is one of the largest WGS interrogations of aTRH to date, the sample size, particularly in the AA and EA strata, was limited due to the extreme nature of this BP trait. Further, we had limited information on white coat HTN, adherence information, and medication doses, which could contribute to phenotype misclassification. While we did observe limited replication for rs12476527 in the BioVU EAs and AAs, we could not directly replicate the results from our multi-ethnic strata due to limitations of the summary statistics available (i.e., no race-combined analyses available *in silico*). Further, most of our novel findings were in the aggregate, gene-based analysis and we did not find another study population with appropriate phenotyping and sequencing data to facilitate replication. In general, studying rare variants linked to drug response introduces a series of challenges regarding the validation of findings both concerning the ascertainment of the replication sample and difficulties identifying the same variants comprising the gene region of interest (Hirschhorn and Altshuler, 2002). Difficulties identifying relevant variants may be further amplified by limited subsamples with specific drug exposures and/or strict inclusion criteria for clinical studies (Aslibekyan et al., 2013). Unfortunately, translation may be limited by the lack of validation in these types of studies. However, the increasing availability of large datasets from diverse biobanks linked to electronic medical records may alleviate current challenges. In conclusion, our main findings included variants in *KCNK3, SCN10A* (a known cardiac gene), *NKAIN2*, and the intergenic region between *ANGPT4-RSPO4*, and aggregate findings in the gene regions of *AGTPBP1, MYL4, PDCD4, BBS9, ERG*, and *IER3*. While many of these SNVs and gene regions have prior cardiovascular implications, most have not been associated with HTN or BP phenotypes (except for *KCNK3*) and remain worthy of further investigation of aTRH.

## Data Availability

Publicly available datasets were analyzed in this study. This data can be found here: The datasets analyzed for this study can be found in the National Center for Biotechnology Information database of Genotypes and Phenotypes (dbGaP) at the following accession numbers: Amish Complex Disease Research Program (AMISH) (phs000956); Atherosclerosis Risk in Communities (ARIC) study (phs001211); The BioMe Biobank at Mount Sinai (BioMe) (phs001644); Cleveland Family Study (CFS) (phs000954); Cardiovascular Health Study (CHS) (phs001368); Genetic Epidemiology Network of Arteriopathy (GENOA) (phs001345); Genetic Epidemiology Network of Saltandhyphen;Sensitivity (GenSALT) (phs001217); Hypertension Genetic Epidemiology Network (HyperGEN) (phs001293); Jackson Heart Study (JHS) (phs000964); Multi-Ethnic Study of Atherosclerosis (MESA) (phs001416. v1. p1); Taiwan Study of Hypertension using Rare Variants (THRV) (phs001387); and Women’s Health Initiative (WHI) (phs001237).

## Group members of TOPMed consortium

Aakrosh Ratan, Adam Szpiro, Adel El Boueiz, Adolfo Correa, Adrienne M. Stilp, Afshin Parsa, Alex Reiner, Allison Ashley-Koch, Alvaro Alonso, Alyna Khan, Amber Beitelshees, Amol Shetty, Andrew Johnson, Aniket Shetty, Ani Manichaikul, Arden Moscati, Auyon Ghosh, Avram Walts, Barbara Konkle, Barry Make, Ben Heavner, Bertha Hidalgo, Beverly Snively, Bonnie Neltner, Bo-Juen Chen, Braxton D. Mitchell, Brian Cade, Brian Custer, Brian Hobbs, Brian Silver, Bruce Gelb, Bruce Psaty, Bruce Weir, C. Charles Gu, Carolina Roselli, Carla Wilson, Carole Sztalryd, Catherine Tong, Cathy Laurie, Carlos Bustamante, Cara Carty, Cashell Jaquish,Caitlin McHugh, Carolyn Crandall, Charles Farber, Chris Gignoux, Chris Frazar, Colleen Davis, Coleen Damcott, Clary Clish, Christy Chang, Chii Min Hwu, Chao (Agnes) Hsiung, Craig Hersh, Craig Johnson, Cecelia Laurie, Charles Kooperberg, Christoph Lange, Cristen Willer, Charles Eaton, Christine Albert, Christine Seidman, Courtney Montgomery, Christopher Scheller, Dandi Qiao, Dan Roden, Dan Arking, David Levine, David Paik, Dan Levy, Daniel Taliun, Daniel Harris, Daniel Grine, Daniel McGoldrick, Da-Wei Gong, David Glahn, Dawood Darbar, Daniel Chasman, David Herrington, David Tirschwell, Danish Saleheen, David Van Den Berg, Daniel E. Weeks, Dawn DeMeo, David Schwartz, Deborah A Meyers, D.C. Rao, Degui Zhi, Dhananjay Vaidya, Deborah Nickerson, Deepti Jain, Donald W. Bowden, Deborah Applebaum-Bowden, Dimitrios Avramopoulos, Donna K Arnett, Diane Becker, Edwin Silverman, Elizabeth Regan, Elizabeth Streeten, Ellen Schmidt, Elliott Hong, Elaine Cornell, Erin Buth, Eric Boerwinkle, Emelia Benjamin, Emily Barron-Casella, Ethan Lange, Ester Cerdeira Sabino, Fei Fei Wang, Francois Aguet, Frank Sciurba, Garrett Storm, George Papanicolaou, Gerald Beck, Greg Kinney, Gonçalo Abecasis, Girish Nadkarni, Harald Goring, Haley Huston, Hao Mei, Heather Ochs-Balcom, Henry Lin, Hemant Tiwari, Heming Wang, Hyun Min Kang, Honghuang Lin, Holly Kramer, Huichun Xu, Hua Tang, Ingo Ruczinski, Ivana Yang, James Hixson, James Luo, James Crapo, James Casella, Jennifer Wessel, Jeff O'Connell, Jeff Haessler, Jeffrey Curtis, JoAnn Manson, John Hokanson, Joanne Curran, Jiang He, Jennifer Brody, Jonathan Cardwell, Jai Broome, John Blangero, Joshua Bis, Jerome Rotter, James Wilson, Jiwon Lee, Jonathon LeFaive, Joshua Lewis, Julia Powers Becker, Joseph Wu, John Barnard, Jonathan Seidman, Julie Mikulla, Jennifer Smith, Jon Peter Durda, Josh Smith, Jessica Lasky Su, Jody Sylvia, Josyf C Mychaleckyj, Jill Johnsen, Juan Manuel Peralta, James Perry, James Pankow, Kevin Sandow, Kathleen Ryan, Kari North, Kathryn Lunetta, Kimberly Jones, Karin Hoth, Kathleen Barnes, Karen Bunting, Keng Han Lin, Karen Schwander, Kent D. Taylor, Karol Watson, Kayleen Williams, Ken Rice, Kristin Ardlie, Lesley Tinker, Lauren Margolin, Lucinda Fulton, Lisa de las Fuentes, L. Adrienne Cupples, Lee-Ming Chuang, Larry Bielak, Lewis Becker, Luisa Mestroni, Lisa Martin, Lucas Barwick, Lynette Ekunwe, Leslie Emery, Leslie Lange, Laura Raffield, Laura RasmussenTorvik, Lu-Chen Weng, L. Keoki Williams, Lisa Yanek, Laura Almasy, Lawrence S Phillips, M M. Benjamin Shoemaker, Michael Mahaney, Min A Jhun, Michael Hall, Mark Gladwin, Mark Geraci, Margery Gass, Mao Fu, Matthew Flickinger, Michael DeBaun, Michelle Daya, Mariza de Andrade, Matthew Conomos, Mina Chung, Michael Cho, Marguerite Ryan Irvin, Mark Chaffin, Marcos Bezerra, Matt Moll, May E. Montasser, Mollie Minear, Myriam Fornage, Merry-Lynn McDonald, Meryl LeBoff, Michael Tsai, Meher Preethi Boorgula, Michael Preuss, Maryam Zekavat, Michael Zody, Machiko Threlkeld, Muagututi’a Sefuiva Reupena, Michael Snyder, Margaret Taub, Matthew Taylor, Marilyn Telen, Marco Perez, Michael Kessler, N Norann Zaghloul, Nora Franceschini, Nicola L. Hawley, Namrata Gupta, Nancy Min, Namiko Abe, Nicholas Smith, Nona Sotoodehnia, Nicholette Palmer, Nicholas Rafaels, Peter VandeHaar, Pradeep Natarajan, Patrick Ellinor, Peter Castaldi, Patrick McArdle, Pamela Russell, Peter Anderson, Pramod Anugu, Patricia Peyser, Pankaj Qasba, Paul Auer, Quenna Wong, Qing Duan, Russell Tracy, Ruth J.F. Loos, Rakhi Naik, Richard Gibbs, Rebecca Jackson, Robert Gerszten, Ranjan Deka, Ren-Hua Chung, Ryan Hernandez, Russell Bowler, Richard Casaburi, Rebecca Beer, Ryan L Minster, Rasika Mathias, Robert Wallace, Rongze Yang, Robert Reed, Rich Johnston, R. Graham Barr, Ravi Duggirala, Robert Kaplan, Steven Salzberg, Shabnam Salimi, Sarah Ruuska, Steven Lubitz, Sharon Graw, Stephanie Gogarten, Soren Germer, Stacey Gabriel, Shanshan Gao, Stephanie M. Fullerton, Scott Devine, Sayantan Das, Sean David, Suzy Comhair, Seung Hoan Choi, Sameer Chavan, Susan Heckbert, Sean McFarland, Solomon Musani, Stanford Mwasongwe, Stephen McGarvey, Susanne May, Susan Mathai, Serpil Erzurum, Susan Dutcher, Simin Liu, Seunggeun Shawn Lee, Simeon Taylor, Stephanie L. Sherman, Sarah Tishkoff, Scott Vrieze, Scott Weiss, Snow Xueyan Zhao, Sebastian Zoellner, Susan Redline, Stephen Rich, Seth Ament, Stella Aslibekyan, Sylvia Smoller, Sergei Nekhai, Sarah C. Nelson, Sharon Kardia, Sekar Kathiresan, Shannon Kelly, Tarik Walker, Tim O’Connor, Take Naseri, Tasha Fingerlin, Thomas Blackwell, Takis Benos, Terri Beaty, Tanja Smith, Timothy A. Thornton, Tamar Sofer, Toni Pollin, Tim Assimes, Ulrike Peters, Ulrich Broeckel, Vasan S. Ramachandran, Vijay G. Sankaran, Vivien Sheehan, Vincent Carey, Wei Zhao, Weiniu Gan, Wendy Post, Wonji Kim, Wayne Hui-Heng Sheu, Wen-Jane Lee, Wei-Min Chen, Xiang Zhou, Xiaofeng Zhu, Xiaohui Li, Xiaoqi (Priscilla) Geng, Xiuqing Guo, Yingze Zhang, Yan Gao, Yongmei Liu, Yi-Cheng Chang, Yii-Der Ida Chen, Yi-Jen Hung, Yue Guan, Yu Liu, Yun Ju Sung, Yun Li, Zhaohui Qin.

## APPENDIX A

Study Specific Acknowledgements

Amish: The TOPMed component of the Amish Research Program was supported by NIH grants R01 HL121007, U01 HL072515, and R01 AG18728.
ARIC: Whole genome sequencing (WGS) for the Trans-Omics in Precision Medicine (TOPMed) program was supported by the National Heart, Lung and Blood Institute (NHLBI). WGS for “NHLBI TOPMed: Atherosclerosis Risk in Communities (ARIC)” (phs001211) was performed at the Baylor College of Medicine Human Genome Sequencing Center (HHSN268201500015C and 3U54HG003273-12S2) and the Broad Institute for MIT and Harvard (3R01HL092577-06S1). Centralized read mapping and genotype calling, along with variant quality metrics and filtering were provided by the TOPMed Informatics Research Center (3R01HL-117626-02S1). Phenotype harmonization, data management, sample-identity QC, and general study coordination, were provided by the TOPMed Data Coordinating Center (3R01HL-120393-02S1). We gratefully acknowledge the studies and participants who provided biological samples and data for TOPMed. The Genome Sequencing Program (GSP) was funded by the National Human Genome Research Institute (NHGRI), the National Heart, Lung, and Blood Institute (NHLBI), and the National Eye Institute (NEI). The GSP Coordinating Center (U24 HG008956) contributed to cross-program scientific initiatives and provided logistical and general study coordination. The Centers for Common Disease Genomics (CCDG) program was supported by NHGRI and NHLBI, and whole genome sequencing was performed at the Baylor College of Medicine Human Genome Sequencing Center (UM1 HG008898 and R01HL059367).
The Atherosclerosis Risk in Communities study has been funded in whole or in part with Federal funds from the National Heart, Lung, and Blood Institute, National Institutes of Health, Department of Health and Human Services (contract numbers HHSN268201700001I, HHSN268201700002I, HHSN268201700003I, HHSN268201700004I and HHSN268201700005I). The authors thank the staff and participants of the ARIC study for their important contributions.
BioMe: The Mount Sinai BioMe Biobank has been supported by The Andrea and Charles Bronfman Philanthropies and in part by Federal funds from the NHLBI and NHGRI (U01HG00638001; U01HG007417; X01HL134588). We thank all participants in the Mount Sinai Biobank. We also thank all our recruiters who have assisted and continue to assist in data collection and management and are grateful for the computational resources and staff expertise provided by Scientific Computing at the Icahn School of Medicine at Mount Sinai.
CHS: This research was supported by contracts HHSN268201200036C, HHSN268200800007C, HHSN268201800001C, N01HC55222, N01HC85079, N01HC85080, N01HC85081, N01HC85082, N01HC85083, N01HC85086, 75N92021D00006, and grants U01HL080295, HL105756, and U01HL130114 from the National Heart, Lung, and Blood Institute (NHLBI), with additional contribution from the National Institute of Neurological Disorders and Stroke (NINDS). Additional support was provided by R01AG023629 from the National Institute on Aging (NIA). A full list of principal CHS investigators and institutions can be found at CHS-NHLBI.org. The content is solely the responsibility of the authors and does not necessarily represent the official views of the National Institutes of Health.
CFS: The Cleveland Family Study has been supported in part by National Institutes of Health grants R01HL046380, KL2RR024990, R35HL135818, and R01HL113338.
GENOA: Support for GENOA was provided by the National Heart, Lung and Blood Institute (HL054457, HL054464, HL054481, HL119443, and HL087660) of the National Institutes of Health.
GenSalt: The Genetic Epidemiology Network of Salt-Sensitivity (GenSalt) was supported by research grants (U01HL072507, R01HL087263, and R01HL090682) from the National Heart, Lung and Blood Institute, National Institutes of Health, Bethesda, MD.
HyperGEN: The HyperGEN Study is part of the National Heart, Lung, and Blood Institute (NHLBI) Family Blood Pressure Program; collection of the data represented here was supported by grants U01 HL054472 (MN Lab), U01 HL054473 (DCC), U01 HL054495 (AL FC), and U01 HL054509 (NC FC). The HyperGEN: Genetics of Left Ventricular Hypertrophy Study was supported by NHLBI grant R01 HL055673 with whole-genome sequencing made possible by supplement -18S1.
JHS: The Jackson Heart Study (JHS) is supported and conducted in collaboration with Jackson State University (HHSN268201800013I), Tougaloo College (HHSN268201800014I), the Mississippi State Department of Health (HHSN268201800015I) and the University of Mississippi Medical Center (HHSN268201800010I, HHSN268201800011I and HHSN268201800012I) contracts from the National Heart, Lung, and Blood Institute (NHLBI) and the National Institute on Minority Health and Health Disparities (NIMHD). The authors also wish to thank the staffs and participants of the JHS. The views expressed in this manuscript are those of the authors and do not necessarily represent the views of the National Heart, Lung, and Blood Institute; the National Institutes of Health; or the U.S. Department of Health and Human Services.
MESA: Whole genome sequencing (WGS) for the Trans-Omics in Precision Medicine (TOPMed) program was supported by the National Heart, Lung and Blood Institute (NHLBI). WGS for “NHLBI TOPMed: Multi-Ethnic Study of Atherosclerosis (MESA)” was performed at the Broad Institute of MIT and Harvard (3U54HG003067-13S1). Centralized read mapping and genotype calling, along with variant quality metrics and filtering were provided by the TOPMed Informatics Research Center (3R01HL-117626-02S1). Phenotype harmonization, data management, sample-identity QC, and general study coordination, were provided by the TOPMed Data Coordinating Center (3R01HL-120393-02S1), and TOPMed MESA Multi-Omics (HHSN2682015000031/HSN26800004). The MESA projects are conducted and supported by the National Heart, Lung, and Blood Institute (NHLBI) in collaboration with MESA investigators. Support for the Multi-Ethnic Study of Atherosclerosis (MESA) projects are conducted and supported by the National Heart, Lung, and Blood Institute (NHLBI) in collaboration with MESA investigators. Support for MESA is provided by contracts 75N92020D00001, HHSN268201500003I, N01-HC-95159, 75N92020D00005, N01-HC-95160, 75N92020D00002, N01-HC-95161, 75N92020D00003, N01-HC-95162, 75N92020D00006, N01-HC-95163, 75N92020D00004, N01-HC-95164, 75N92020D00007, N01-HC-95165, N01-HC-95166, N01-HC-95167, N01-HC-95168, N01-HC-95169, UL1-TR-000040, UL1-TR-001079, UL1-TR-001420, UL1TR001881, DK063491, and R01HL105756. The authors thank the other investigators, the staff, and the participants of the MESA study for their valuable contributions. A full list of participating MESA investigators and institutes can be found at http://www.mesa-nhlbi.org.
THRV: The Rare Variants for Hypertension in Taiwan Chinese (THRV) is supported by the National Heart, Lung, and Blood Institute (NHLBI) grant (R01HL111249) and its participation in TOPMed is supported by an NHLBI supplement (R01HL111249-04S1). THRV is a collaborative study between Washington University in St. Louis, LA BioMed at Harbor UCLA, University of Texas in Houston, Taichung Veterans General Hospital, Taipei Veterans General Hospital, Tri-Service General Hospital, National Health Research Institutes, National Taiwan University, and Baylor University. THRV is based (substantially) on the parent SAPPHIRe study, along with additional population-based and hospital-based cohorts. SAPPHIRe was supported by NHLBI grants (U01HL54527, U01HL54498) and Taiwan funds, and the other cohorts were supported by Taiwan funds.
WHI: The WHI program is funded by the National Heart, Lung, and Blood Institute, National Institutes of Health, U.S. Department of Health and Human Services through contracts HHSN268201600018C, HHSN268201600001C, HHSN268201600002C, HHSN268201600003C, and HHSN268201600004C. The authors thank the WHI investigators and staff for their dedication, and the study participants for making the program possible. A full listing of WHI investigators can be found at: https://www-whi-org.s3.us-west-2.amazonaws.com/wp-content/uploads/WHI-Investigator-Long-List.pdf.
BioVU: The dataset(s) used for analyses described were obtained from Vanderbilt University Medical Center’s BioVU, which is supported by numerous sources: institutional funding, private agencies, and federal grants. These include the NIH funded Shared Instrumentation Grant S10RR025141; and CTSA grants UL1TR002243, UL1TR000445, and UL1RR024975. Genomic data are also supported by investigator-led projects that include U01HG004798, R01NS032830, RC2GM092618, P50GM115305, U01HG006378, U19HL065962, R01HD074711; and additional funding sources listed at https://victr.vumc.org/biovu-funding/.
