# Supplementary material for: Whole genome sequence analysis of apparent treatment resistant hypertension status in participants from the Trans-Omics for Precision Medicine program
Source: Front Genet. 2023 Dec 13;14:1278215. doi: 10.3389/fgene.2023.1278215 (PMC10755672; doi:10.3389/fgene.2023.1278215)
Supplement: Supplementary file 1 [file DataSheet1.pdf]

Supplementary Material

Nicole D. Armstrong[1*], Vinodh Srinivasasainagendra[2], Farah Ammous[3,4], Themistocles L. Assimes[5], Amber L. Beitelshees[6], Jennifer Brody[7], Brian E. Cade[8], Yii-Der Ida Chen[9], Han Chen[10,11], Paul S. de Vries[10], James S. Floyd[12,13], Nora Franceschini[14], Xiuqing Guo[9], Jacklyn N. Hellwege[15,16], John S. House[17], Chii-Min Hwu[18], Sharon LR Kardia[3], Ethan M. Lange[19], Leslie A. Lange[19], Caitrin W. McDonough[20], May E. Montasser[6], Jeffrey R. O'Connell[21], Megan M. Shuey[15,16], Xiao Sun[22], Rikki M. Tanner[1], Zhe Wang[23], Wei Zhao[3,4], April P. Carson[24], Todd L. Edwards[16,25], Tanika N. Kelly[26], Eimear E. Kenny[27], Charles Kooperberg[28], Ruth J.F. Loos[23], Alanna C. Morrison[10], Alison Motsinger-Reif[17], Bruce M. Psaty[7,29,30], Dabeeru C. Rao[31], Susan Redline[8], Stephen S. Rich[32], Jerome I. Rotter[9], Jennifer A. Smith[3,4], Albert V. Smith[33], Marguerite R. Irvin[1], Donna K. Arnett[34], on behalf of the NHLBI Trans-Omics for Precision Medicine (TOPMed) Consortium and the TOPMed Blood Pressure Working Group

[1]Department of Epidemiology, University of Alabama at Birmingham, Birmingham AL, USA
[2]Department of Biostatistics, University of Alabama at Birmingham, Birmingham AL, USA
[3]Department of Epidemiology, School of Public Health, University of Michigan, Ann Arbor, MI, USA
[4]Survey Research Center, Institute for Social Research, Ann Arbor, MI, USA
[5]Division of Cardiovascular Medicine, Department of Medicine, Stanford University School of Medicine, Palo Alto, CA, USA
[6]Division of Endocrinology, Diabetes, and Nutrition, Program for Personalized and Genomic Medicine, Department of Medicine, University of Maryland School of Medicine, Baltimore, MD, US
[7]Cardiovascular Health Research Unit, Department of Medicine, University of Washington, Seattle, WA, USA
[8]Division of Sleep and Circadian Disorders, Department of Medicine, Brigham and Women's Hospital, Boston, MA, USA
[9] The Institute for Translational Genomics and Population Sciences, Department of Pediatrics, The Lundquist Institute for Biomedical Innovation at Harbor-UCLA Medical Center, Torrance, CA USA
[10]Human Genetics Center, Department of Epidemiology, Human Genetics, and Environmental Sciences, School of Public Health, The University of Texas Health Science Center at Houston, Houston, TX, USA
[11]Center for Precision Health, School of Biomedical Informatics, The University of Texas Health Science Center at Houston, Houston, TX, USA
[12]Department of Medicine, University of Washington, Seattle, WA, USA
[13]Department of Epidemiology, University of Washington, Seattle, WA, USA
[14]Department of Epidemiology, Gillings School of Global Public Health, University of North Carolina, Chapel Hill, NC, USA
[15]Division of Genetic Medicine, Department of Medicine, Vanderbilt University Medical Center, Nashville, TN, USA
[16]Vanderbilt Genetics Institute, Vanderbilt University Medical Center, Nashville, TN, USA
[17]Biostatistics and Computational Biology Branch, National Institute of Environmental Health Sciences, Durham, NC, USA
[18] Section of Endocrinology and Metabolism, Department of Medicine, Taipei Veterans General Hospital, Taipei, Taiwan

[19]Division of Biomedical Informatics and Personalized Medicine, Department of Medicine, University of Colorado Anschutz Medical Campus, Aurora, CO, USA
[20]Department of Pharmacotherapy and Translational Research, University of Florida, College of Pharmacy, Gainesville, FL, USA
[21]Department of Medicine, University of Maryland, Baltimore, MD, USA
[22]Department of Epidemiology, Tulane University School of Public Health and Tropical Medicine, New Orleans, LA, USA
[23]The Charles Bronfman Institute for Personalized Medicine, Icahn School of Medicine at Mount Sinai, New York, NY, USA
[24]Department of Medicine, University of Mississippi Medical Center, Jackson, MS, USA
[25]Division of Epidemiology, Department of Medicine, Vanderbilt University Medical Center, Nashville, TN, USA
[26]Division of Nephrology, Department of Medicine, College of Medicine, University of Illinois Chicago, Chicago, IL, USA
[27]Institute for Genomic Health, Icahn School of Medicine at Mount Sinai, New York, NY, USA
[28]Division of Public Health Sciences, Fred Hutchinson Cancer Research Center, Seattle, WA, USA
[29]Department of Epidemiology, University of Washington, Seattle, WA, USA
[30]Department of Medicine, University of Washington, Seattle, WA, USA
[31]Division of Biostatistics, School of Medicine, Washington University in St. Louis, St. Louis, MO, USA
[32]Department of Public Health Sciences, Center for Public Health Genomics, University of Virginia, Charlottesville, VA, USA
[33]Center for Statistical Genetics, Department of Biostatistics, University of Michigan School of Public Health, Ann Arbor, MI, USA
[34]Office of the Provost, University of South Carolina, Columbia, SC, USA

Study Populations

The Amish Complex Disease Research Program (Amish): The Amish study includes a set of large community-based studies focused largely on cardiometabolic health carried out in the Old Order Amish (OOA) community of Lancaster County, Pennsylvania, where over 7,000 Amish have been recruited to date. The Amish cohort participating in the Trans-Omics for Precision Medicine (TOPMed) Consortium comprises 1,120 subjects ≥ 18 years of age from large multigenerational families who were recruited for specific protocols between 2001 and 2006 [1]. BP was measured at baseline in triplicate using a standard sphygmomanometer, with the average of the second and third measurement being used for analysis. The current study includes 947 participants (3 aTRH cases, 29 treatment responsive controls, and 915 normotensive controls).

Atherosclerosis Risk in Communities (ARIC) Study: The ARIC study is a population-based prospective cohort study of cardiovascular disease sponsored by the National Heart, Lung, and Blood Institute (NHLBI). ARIC included 15,792 individuals, predominantly European American and African American, aged 45-64 years at baseline (1987-89), chosen by probability sampling from four US communities. Cohort members completed three additional triennial follow-up examinations, a fifth exam in 2011-2013, a sixth exam in 2016-2017, and a seventh exam in 2018-2019. The ARIC study has been described in detail previously [2]. BP was measured using a standardized Hawksley random-zero mercury column sphygmomanometer with participants in

a sitting position after a resting period of 5 minutes. The size of the cuff was chosen according to the arm circumference. Three sequential recordings for systolic and diastolic BP were obtained from the baseline exam, where the mean of the last two measurements was used in this study. BP lowering medication use was recorded from the medication history at the baseline exam. In this study, 6,311 participants (18% African ancestry; 82% European ancestry) were included with relevant phenotype information.

The Mount Sinai BioMe Biobank (BioMe): BioMe Biobank is an ongoing, prospective, hospital- and outpatient- based population research program operated by The Charles Bronfman Institute for Personalized Medicine at Mount Sinai. BioMe enrolled over 50,000 participants between September 2007 and July 2019. BioMe is an Electronic Medical Record (EMR)-linked biobank that integrates research data and clinical care information for consented patients at The Mount Sinai Medical Center, which serves diverse local communities of upper Manhattan with broad health disparities. BioMe populations include African ancestry, Hispanic Latino ancestry, White European ancestry, as well as other ancestries. Information on anthropometrics, demographics, BP and use of BP-lowering medication was derived from participants' EMR. Full genetic and phenotype data for 4,254 was utilized in this study.

The Cleveland Family Study (CFS): The CFS is a family-based cohort study that aims to examine the genetic and familial basis of sleep apnea [3]. The total cohort consists of 2,534 African- and European-American individuals from 356 families. 275 index probands with confirmed sleep apnea diagnoses were recruited from northern Ohio sleep centers. Neighborhood control probands with at least two living relatives available for study were also selected at random from a list provided by the index family. The spouses and relatives of the cases and controls were also recruited if available. Data was collected from up to four visits made from 1990 to 2006, and a final laboratory visit at a clinical research center between 2000 and 2006. Blood samples were obtained from individuals who participated in the final two exam cycles, and DNA was extracted from samples that passed quality control. Two to three measurements of systolic and diastolic BP were obtained from the last available examination for each participant. BP measurements were taken in the sitting or supine positions following standardized guidelines and using a calibrated sphygmomanometer. The average of two BP measures were used in the current analyses. When three BP measures were available for a study participant, the first measure was discarded, and the final two measures were averaged. Medication information was obtained via a standardized questionnaire during exam visits. In the current study, 641 participants (16 cases, 57 treatment responsive controls, 568 normotensive controls) were included.

The Cardiovascular Health Study (CHS): The CHS is a population-based cohort study of risk factors for coronary heart disease and stroke in adults ≥65 years conducted across four field centers [4]. The original cohort of 5,201 predominately European persons was recruited in 1989-1990 from random samples of the Medicare eligibility lists; subsequently, an additional predominantly African American cohort of 687 persons was enrolled for a total sample of 5,888. A subset of 3,622 CHS participants were selected for the TOPMed sequencing program. Blood samples were drawn from all participants at their baseline examination and DNA was subsequently extracted from available samples. Research staff with central training in BP measurement assessed two right-arm systolic and diastolic BP levels at baseline with a Hawksley

random-zero sphygmomanometer. Measures were taken after five minutes of seated rest, with BP measurements taken one minute apart. Means of the two repeated BP measurements from the baseline examination were used for the analyses. Antihypertensive (AHT) use was assessed by medication inventory. [5] A total of 1,712 CHS participants were included in the current aTRH study.

Genetic Epidemiology Network of Arteriopathy (GENOA): The GENOA study consists of hypertensive sibships that were recruited for linkage and association studies in order to identify genes that influence BP and its target organ damage[6, 7]. In Phase I (1996-2001), all members of sibships containing two or more individuals with essential hypertension clinically diagnosed before age 60 were invited to participate, including both hypertensive and normotensive siblings. A total of 1,583 non-Hispanic whites from Rochester, MN, and 1,854 African Americans from Jackson, MS, were examined. Sitting systolic and diastolic BP (mmHg) were measured three times with a random zero sphygmomanometer. The average of the last two measurements at Phase I was used in this study. A physician specializing in hypertension reviewed all medications and made the final determination of whether a medication was considered an AHT. Only African Americans were sequenced through the TOPMed project, and participants who were also in the ARIC or Jackson Heart Study (JHS) were excluded from analysis. A total of 577 African American participants were included in the current analysis.

Genetic Epidemiology Network of Salt-Sensitivity (GenSalt): The GenSalt study is a family feeding-study designed to examine the interaction between genes and dietary sodium and potassium intake on BP [8]. Briefly, 3,142 participants from 633 Han families from rural, north China were ascertained through a proband with untreated pre-hypertension or stage-1 hypertension identified from a population-based BP screening. A total of 1,818 GenSalt probands took part in the TOPMed WGS program. All BP readings were measured by trained and certified observers using a random–zero sphygmomanometer and a standard protocol. BP was measured with the participant in the sitting position after 5 minutes of rest. Systolic and diastolic BP measures were taken in triplicate during each day of a three-day baseline observation. After throwing out the first measure, the subsequent two measures obtained on the first day of baseline observation were averaged and used in this analysis. 1,608 GenSalt participants were utilized as normotensive controls.

Hypertension Genetic Epidemiology Network (HyperGEN): The HyperGEN study is one of the four networks in the Family Blood Pressure Program (FBPP) supported by the NHLBI to identify genetic contributors to hypertension [9]. HyperGEN is a family-based study with a sib-pair design. African American sibships with hypertension were recruited from Forsyth County, NC and from the community-at-large in Birmingham, AL from 1995 to 2000, where sib-pairs with hypertension onset before age 60 were recruited in Phase I. Demographic information, current medications, co-morbid conditions, and clinical measures were collected during the study visit. The study was subsequently extended to siblings and the offspring of the hypertensive probands who were unmedicated adults. BP was measured with an automated oscillometric device using a standard protocol. After a 5-minute resting period, BP was measured six times, with each BP measurement taken two minutes apart. The average of the second and third BP

measurements was averaged and used in this analysis. A total of 911 HyperGEN participants were included in the current analysis.

Jackson Heart Study (JHS): The JHS is a large, population-based observational study evaluating the etiology of cardiovascular, renal, and respiratory diseases among African Americans residing in the three counties that make up the Jackson, Mississippi metropolitan area [10]. Data, including medical history, and biologic materials have been collected from 5306 participants, including biochemical measures and diagnostic procedures during a baseline examination (2000-2004) and two follow-up examinations (2005-2008 and 2009-2012). Participants presented all medications used within two weeks whether prescriptions or over the counter during the clinical visit. The Medi-Span® therapeutic classification system was used to identify medication. Samples for genomic DNA were collected during the first two examinations and WGS data are available for 3,307 participants. SBP and DBP were measured twice after the participant had been seated for 5 minutes. BP was measured using a Hawksley random-zero sphygmomanometer and the average of the two BP measures were used for the current analysis. There were 1,853 JHS participants, including 168 aTRH cases, included in the current study.

Multi-ethnic Study of Atherosclerosis (MESA): MESA is a study of the characteristics of subclinical cardiovascular disease and the risk factors that predict progression to clinically overt cardiovascular disease or progression of the subclinical disease [11]. MESA consisted of a diverse (African, European, Hispanic, and Asian), community-based sample of 6,814 men and women aged 45-84 without known clinical cardiovascular disease, recruited from six field centers across the United States. During clinic visits, BP was measured after a 5-minute rest, and was measured three times at one-minute intervals using a Dinamap PRO 100 automated oscillometric device (Critikon, Tampa, FL) with the subject in seated. The average of the second and third BP measurements was recorded for each visit. Antihypertensive medication use was recorded. A total of 3,535 MESA participants with available WGS and BP data were included in the current analysis.

Taiwan Study of Hypertension using Rare Variants (THRV): The THRV-TOPMed study consists of three participating cohorts: The SAPPHIRe Family Cohort, Tri-Service General Hospital (TSGH, a hospital-based cohort), and the Taichung Veterans General Hospital (TCVGH, a hospital-based cohort), all based in Taiwan. THRV proposed to identify rare and low frequency genetic variants for BP and hypertension through whole exome sequencing of a subset of highly enriched Taiwan Chinese hypertensive families (N=1,200) previously recruited as part of the NHLBI-sponsored SAPPHIRe Network and matched controls. The SAPPHIRe families were recruited to have multiple hypertensive sibs and/or one normotensive/hypotensive sib [12]. For SAPPHIRe, eligible families were sent detailed questionnaires beforehand on medical history, family history, age, sex, physical activity and drinking habits. The TSHG and TCVGH hospital-based cohorts were recruited following comparable inclusion/exclusion criteria and matched SAPPHIRe participants for age, sex, and BMI category. TCVGH utilized extensive electronic medical records (EMR) for information regarding medical history, while TSGH recruited their participants as a traditional study and utilized standardized questionnaires. Resting BP was measured in triplicate using a standard protocol and automated DINAMAP device. The

average of the second and third BP readings were averaged and used in the current analysis. In the current study, 548 THRV participants had relevant phenotype and WGS data.

WHI (Women's Health Initiative): WHI is a national health study that focuses on strategies for preventing common diseases such as heart disease, cancer and fracture in postmenopausal women. A total of 161,808 women aged 50–79 years old were recruited from 40 clinical centers in the US between 1993 and 1998 [13, 14]. The WHI contribution of TOPMed was designed as a case-control study of stroke and venous thromboembolism (VTE). Approximately 5,000 stroke and 1,000 VTE cases and 5,000 controls were selected for WGS. The controls were selected to frequency match the cases by age at recruitment, ethnicity, and membership in the WHI hormone therapy trial. Self-reported medications were collected using standardized questionnaires at the screening visit and BP was measured in a clinical visit by certified staff using standardized procedures and instruments. Two BP measures were recorded after 5 minutes rest using a mercury sphygmomanometer. The average of the two measurements, obtained 30 seconds apart, was used in analyses. A total of 6,311 WHI participants with WGS and relevant BP data were included in the current analysis.

Replication

BioVU: The BioVU DNA Repository is a deidentified database of EMR that are linked to patient DNA samples at Vanderbilt University Medical Center. A detailed description of the database and how it is maintained is described elsewhere. [15] DNA samples were genotyped on a custom Illumina Multi-Ethnic Genotyping Array (Illumina Inc., San Diego, CA) and samples were excluded with missingness rates above 2%, if consent was revoked, sample was duplicated, or failed sex concordance checks. The genotyped data was imputed on the Michigan Imputation Server to the TOPMed r2 reference panel [16] using Minimac4. Among BioVU participants, unrelated self-reported non-Hispanic Black and White individuals ≥18 were selected. A previously published definition of aTRH was utilized and all data was extracted prior to August of 2019 [17]. Briefly, aTRH status was defined through presence of an HTN ICD-9 or ICD-10 code, treatment with an AHT, or having two outpatient, non-emergency department SBP >140 mmHg and/or two DBP measures >90 mmHg. Patients with aTRH were identified based on failure to achieve controlled BP on three AHT, including a thiazide diuretic or prescribed four or more medications regardless of achieving control. The comparison group of hypertensive patients who achieved BP control on one or two medications, excluding participants with chronic kidney disease (stage 4 and 5), or those with secondary causes of HTN. A second control group consisted of individuals without HTN as defined above. Data were stratified by reported race. Across each stratum, logistic regression models analyzing additive variant associations were performed, adjusting for age, age-squared, body mass index, sex, and top 10 genetic principal components. Inferences were limited to genotyped and imputed variants with info quality scores ≥0.4, Hardy-Weinberg equilibrium p-values >5.00E-08, and minor allele counts of ≥20. For our analyses, BioVU provided a look-up of suggestive variants, from >61,000 participants, identified in **Table 2.**

International Consortium of Antihypertensive Pharmacogenomic Studies (ICAPS): Cohorts that participated in ICAPS aTRH analyses included the Action to Control Cardiovascular Risk in Diabetes (ACCORD) trial, Anglo-Scandinavian Cardiac Outcomes Trial (ASCOT), International Verapamil S-Trandolapril Study (INVEST), and the Genetics of Hypertension Associated

Treatments (GenHAT) study, for a total of 10,801 participants (4,494 cases; ~25% non-European). All cohorts excluded individuals with baseline estimated glomerular filtration rate <30 ml/min/1.73 m^2. aTRH was defined as uncontrolled HTN using three or more AHTs from different classes (BP ≥140/90 mmHg) or controlled HTN on four or more AHT medications. Treatment responsive controls were individuals with controlled BP (<140/90) with the use of three or fewer AHTs. Quality control and analyses were performed at the cohort-level for the five randomized controlled clinical trials, and then meta-analyzed (combined and race-stratified). For each cohort, genotyped variants were imputed to the 1000 Genomes Phase 3 reference panel. The additive genetic model assessed examined variant main effect with logistic regression of the log(odds) of aTRH, adjusting for age, sex, BMI, and genetic principal components. For our analyses, ICAPS provided a look-up of suggestive variants identified in **Table 2.**

Supplementary Figures

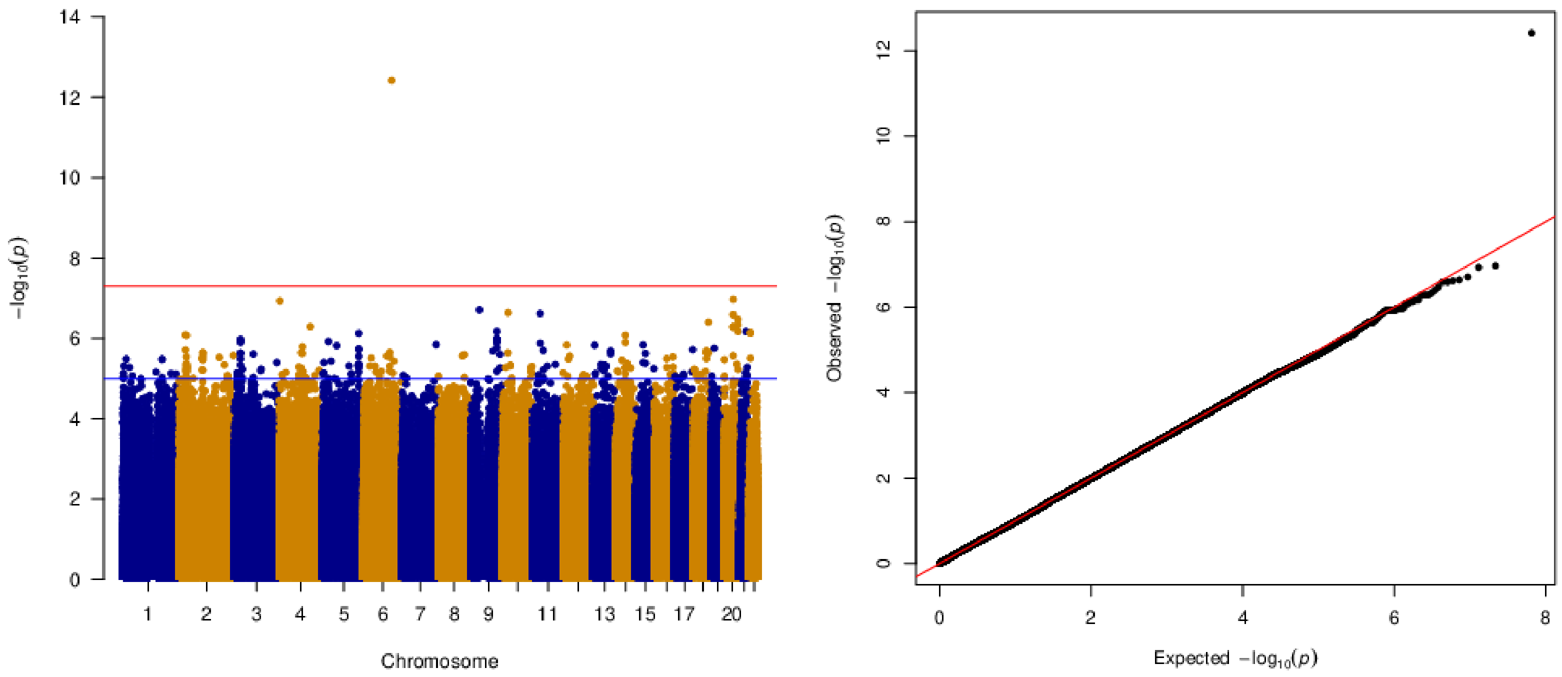

Supplemental Figure S1. Manhattan and QQ plot of aTRH versus normotensive controls in the multi-ethnic analysis.

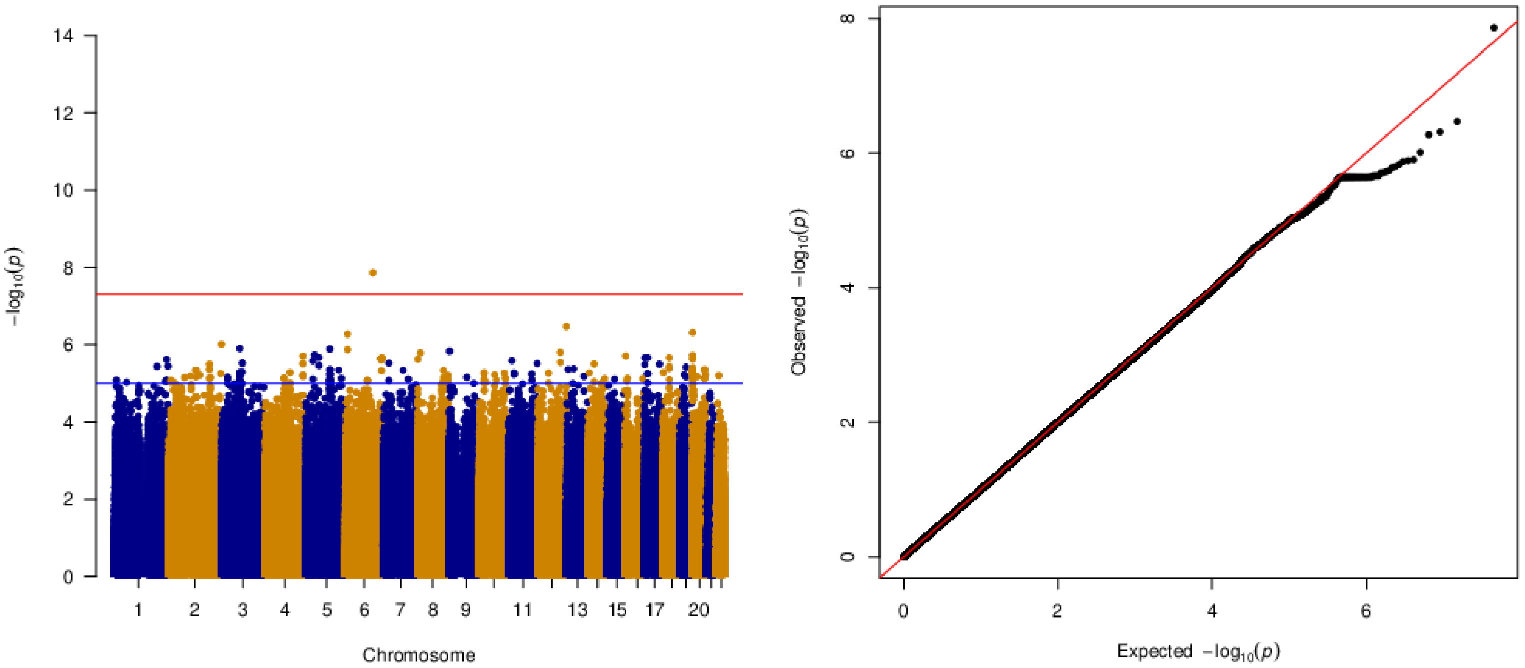

Supplemental Figure S2. Manhattan and QQ plot of aTRH versus treatment-responsive controls in the multi-ethnic analysis.

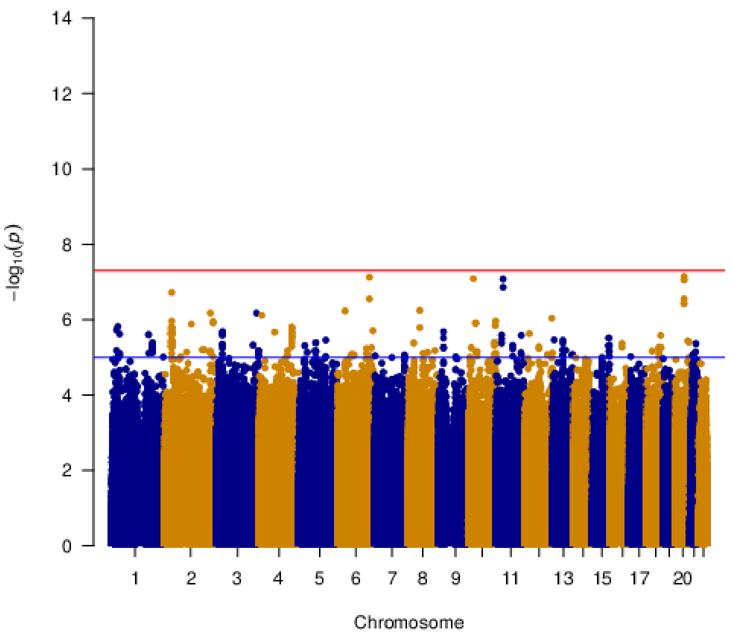

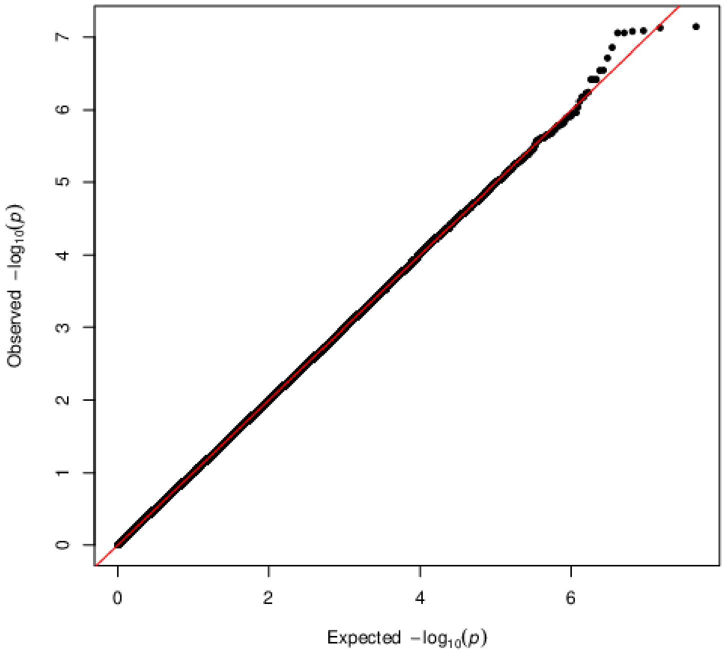

Supplemental Figure S3. Manhattan and QQ plot of aTRH versus normotensive controls in the African-ancestry analysis.

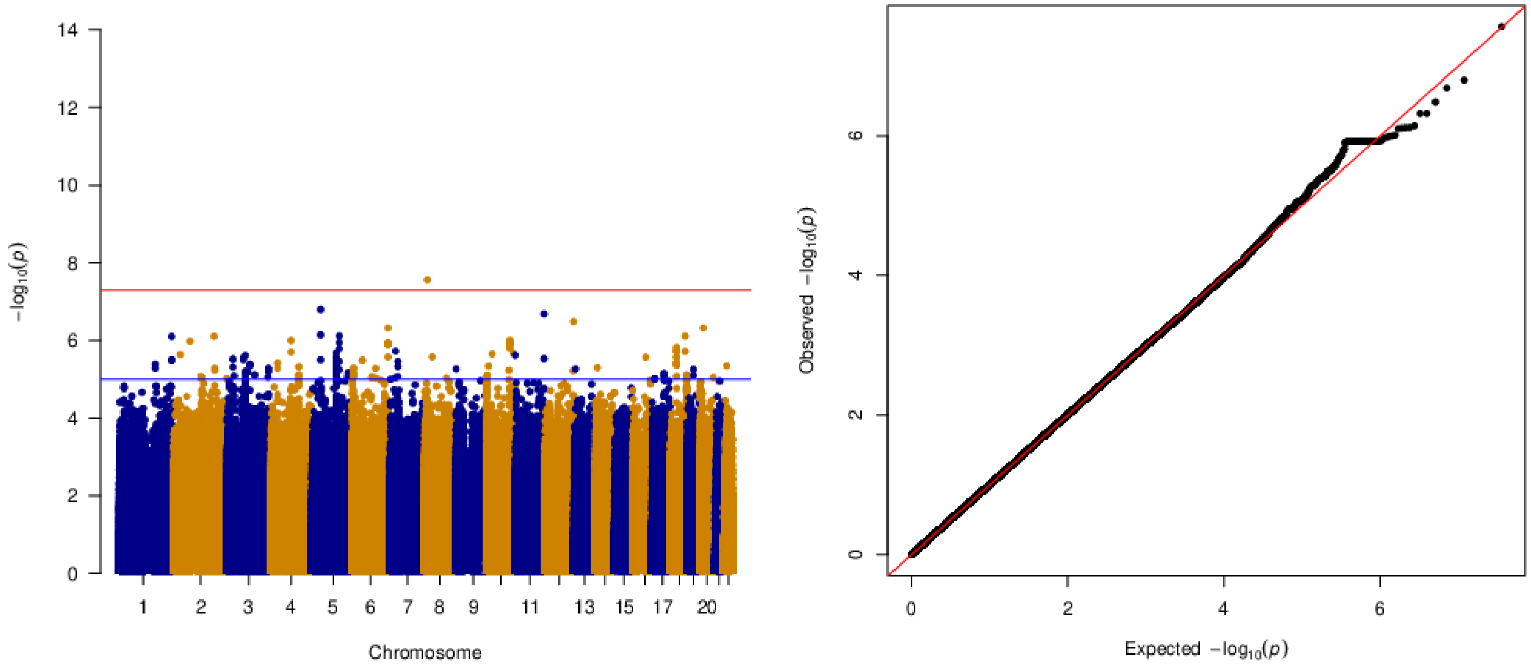

Supplemental Figure S4. Manhattan and QQ plot of aTRH versus treatment-responsive controls in the African-ancestry analysis.

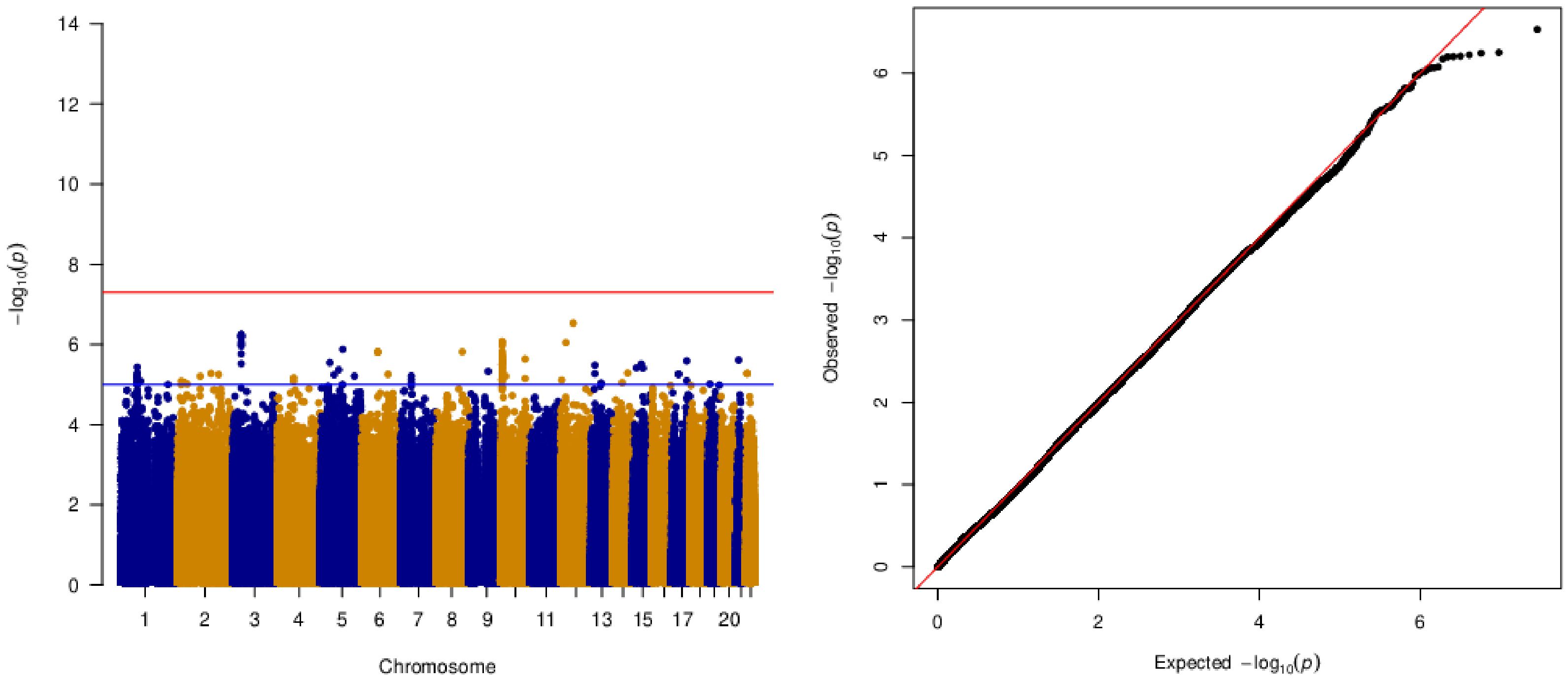

Supplemental Figure S5. Manhattan and QQ plot of aTRH versus normotensive controls in the European-ancestry analysis.

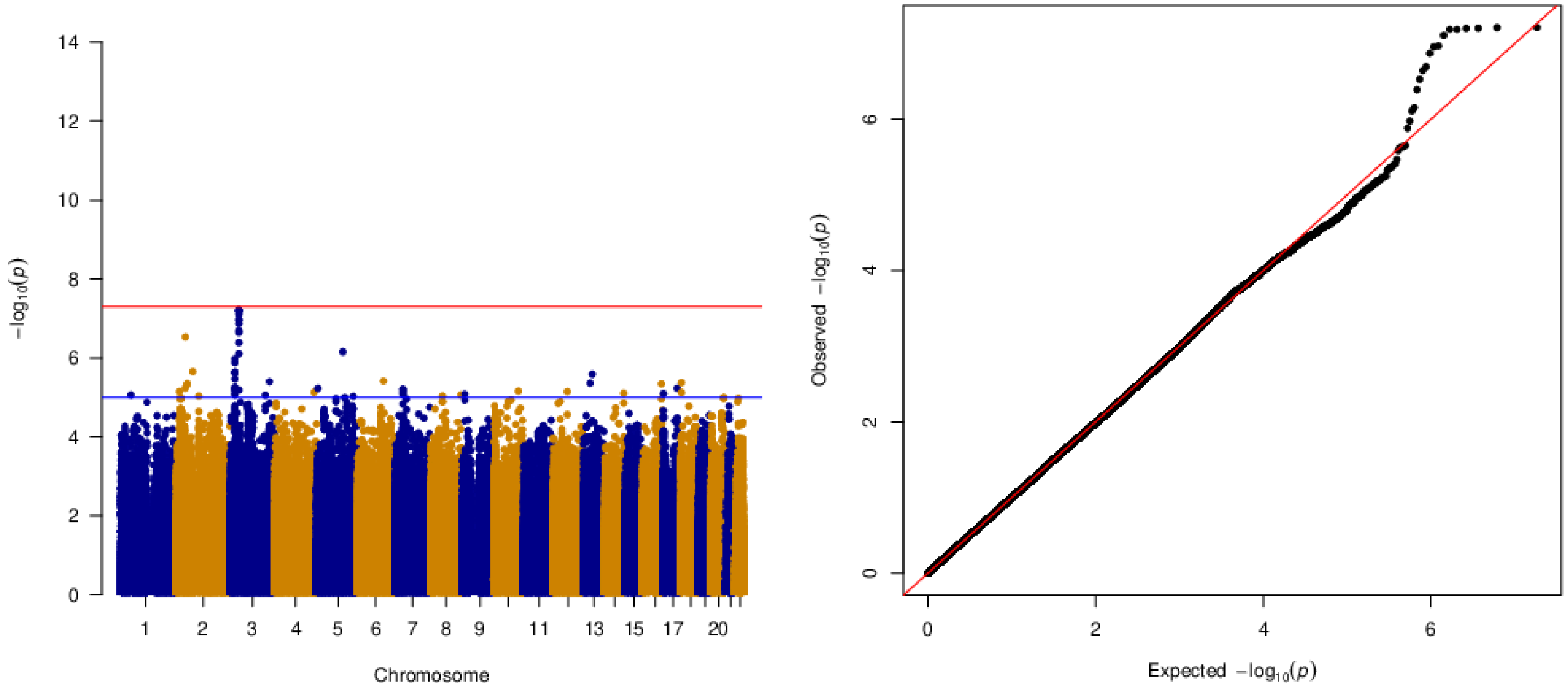

Supplemental Figure S6. Manhattan and QQ plot of aTRH versus treatment-responsive controls in the European-ancestry analysis.

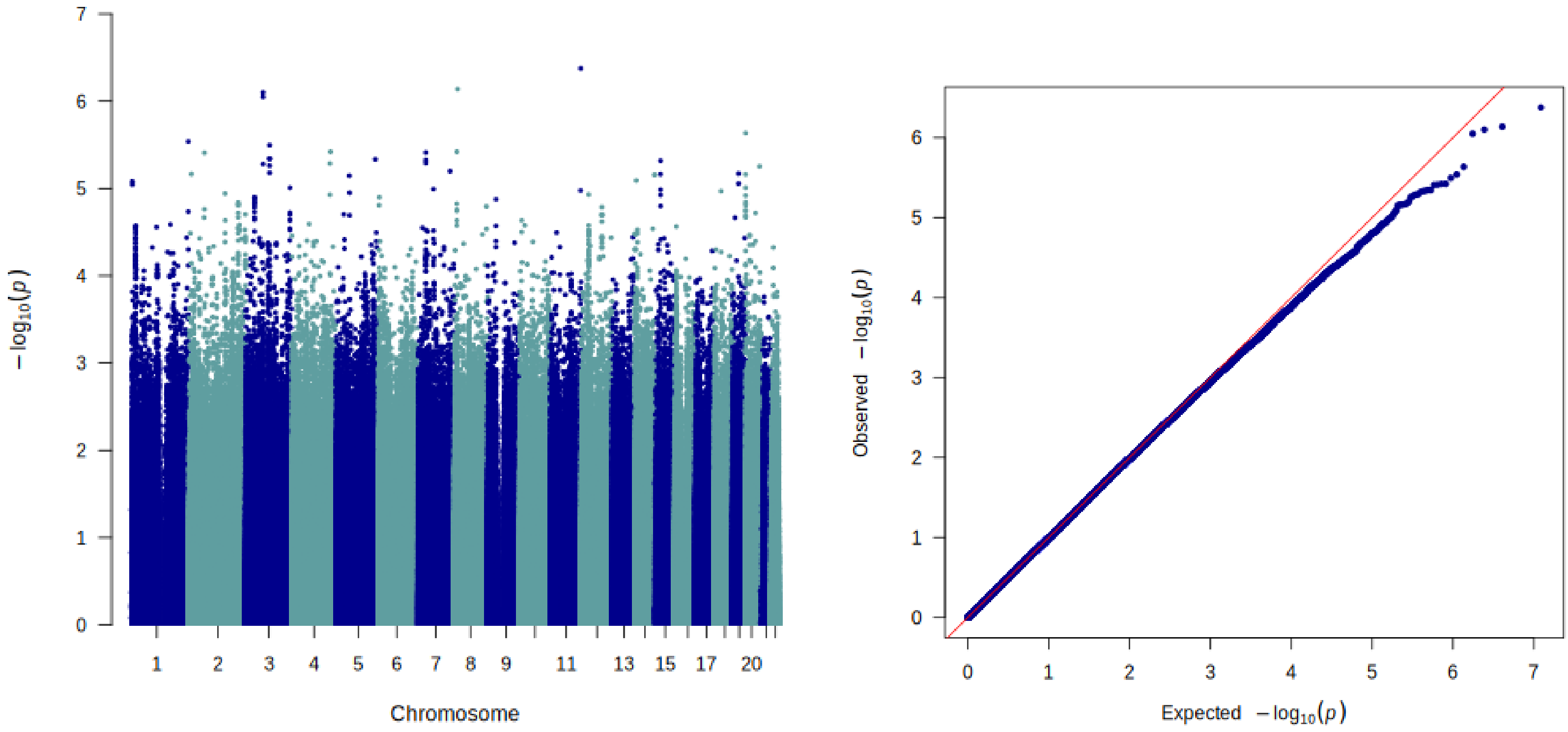

Supplemental Figure S7. Manhattan and QQ plot of meta-analysis for aTRH versus treatment-responsive controls.

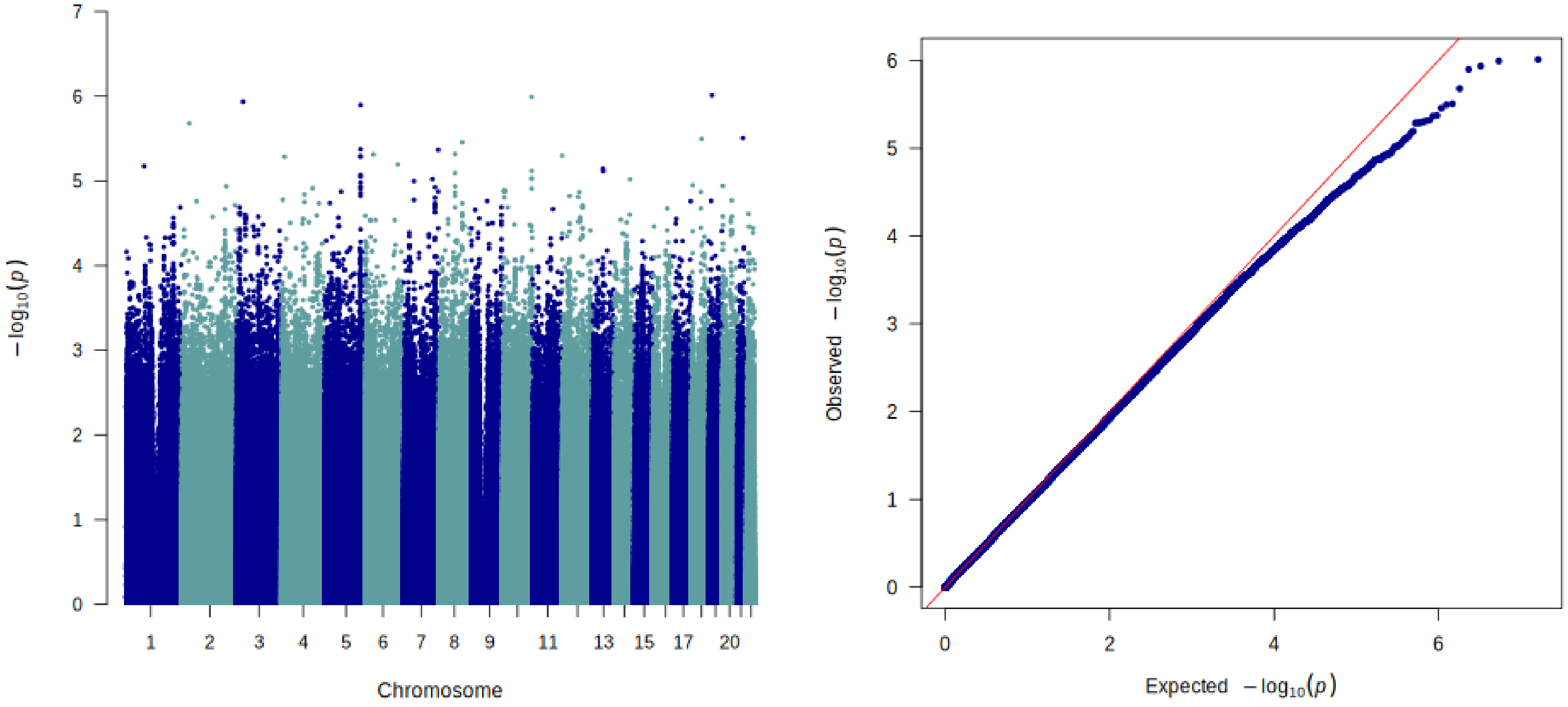

Supplemental Figure S8. Manhattan and QQ plot of meta-analysis for aTRH versus normotensive controls.

Supplementary References

1. Mitchell BD, McArdle PF, Shen H, Rampersaud E, Pollin TI, Bielak LF, et al. The genetic response to short-term interventions affecting cardiovascular function: rationale and design of the Heredity and Phenotype Intervention (HAPI) Heart Study. Am Heart J. 2008;155(5):823-8.
2. The Atherosclerosis Risk in Communities (ARIC) Study: design and objectives. The ARIC investigators. Am J Epidemiol. 1989;129(4):687-702.
3. Redline S, Tishler PV, Tosteson TD, Williamson J, Kump K, Browner I, et al. The familial aggregation of obstructive sleep apnea. Am J Respir Crit Care Med. 1995;151(3 Pt 1):682-7.
4. Fried LP, Borhani NO, Enright P, Furberg CD, Gardin JM, Kronmal RA, et al. The Cardiovascular Health Study: design and rationale. Ann Epidemiol. 1991;1(3):263-76.
5. Psaty BM, Lee M, Savage PJ, Rutan GH, German PS, Lyles M. Assessing the use of medications in the elderly: methods and initial experience in the Cardiovascular Health Study. The Cardiovascular Health Study Collaborative Research Group. J Clin Epidemiol. 1992;45(6):683-92.
6. Investigators F. Multi-center genetic study of hypertension: The Family Blood Pressure Program (FBPP). Hypertension. 2002;39(1):3-9.
7. Daniels PR, Kardia SL, Hanis CL, Brown CA, Hutchinson R, Boerwinkle E, et al. Familial aggregation of hypertension treatment and control in the Genetic Epidemiology Network of Arteriopathy (GENOA) study. Am J Med. 2004;116(10):676-81.
8. GenSalt Collaborative Research G. GenSalt: rationale, design, methods and baseline characteristics of study participants. J Hum Hypertens. 2007;21(8):639-46.
9. Williams RR, Rao DC, Ellison RC, Arnett DK, Heiss G, Oberman A, et al. NHLBI family blood pressure program: methodology and recruitment in the HyperGEN network. Hypertension genetic epidemiology network. Ann Epidemiol. 2000;10(6):389-400.
10. Taylor HA, Jr., Wilson JG, Jones DW, Sarpong DF, Srinivasan A, Garrison RJ, et al. Toward resolution of cardiovascular health disparities in African Americans: design and methods of the Jackson Heart Study. Ethn Dis. 2005;15(4 Suppl 6):S6-4-17.
11. Bild DE, Bluemke DA, Burke GL, Detrano R, Diez Roux AV, Folsom AR, et al. Multi-Ethnic Study of Atherosclerosis: objectives and design. Am J Epidemiol. 2002;156(9):871-81.
12. Ranade K, Hinds D, Hsiung CA, Chuang LM, Chang MS, Chen YT, et al. A genome scan for hypertension susceptibility loci in populations of Chinese and Japanese origins. Am J Hypertens. 2003;16(2):158-62.
13. Design of the Women's Health Initiative clinical trial and observational study. The Women's Health Initiative Study Group. Control Clin Trials. 1998;19(1):61-109.
14. Hays J, Hunt JR, Hubbell FA, Anderson GL, Limacher M, Allen C, et al. The Women's Health Initiative recruitment methods and results. Ann Epidemiol. 2003;13(9 Suppl):S18-77.
15. Roden DM, Pulley JM, Basford MA, Bernard GR, Clayton EW, Balser JR, et al. Development of a large-scale de-identified DNA biobank to enable personalized medicine. Clin Pharmacol Ther. 2008;84(3):362-9.
16. Das S, Forer L, Schonherr S, Sidore C, Locke AE, Kwong A, et al. Next-generation genotype imputation service and methods. Nat Genet. 2016;48(10):1284-7.
17. Shuey MM, Gandelman JS, Chung CP, Nian H, Yu C, Denny JC, et al. Characteristics and treatment of African-American and European-American patients with resistant hypertension

identified using the electronic health record in an academic health centre: a case-control study. BMJ Open. 2018;8(6):e021640.
